# Review of the Early–Middle Pleistocene boundary and Marine Isotope Stage 19

**DOI:** 10.1186/s40645-021-00439-2

**Published:** 2021-09-03

**Authors:** Martin J. Head

**Affiliations:** Department of Earth Sciences, Brock University, St. Catharines, Ontario L2S 3A1 Canada

**Keywords:** Early–Middle Pleistocene, Quaternary, GSSP, MIS 19, Chiba

## Abstract

The Global Boundary Stratotype Section and Point (GSSP) defining the base of the Chibanian Stage and Middle Pleistocene Subseries at the Chiba section, Japan, was ratified on January 17, 2020. Although this completed a process initiated by the International Union for Quaternary Research in 1973, the term Middle Pleistocene had been in use since the 1860s. The Chiba GSSP occurs immediately below the top of Marine Isotope Substage (MIS) 19c and has an astronomical age of 774.1 ka. The Matuyama–Brunhes paleomagnetic reversal has a directional midpoint just 1.1 m above the GSSP and serves as the primary guide to the boundary. This reversal lies within the Early–Middle Pleistocene transition and has long been favoured to mark the base of the Middle Pleistocene. MIS 19 occurs within an interval of low-amplitude orbital eccentricity and was triggered by an obliquity cycle. It spans two insolation peaks resulting from precession minima and has a duration of ~ 28 to 33 kyr. MIS 19c begins ~ 791–787.5 ka, includes full interglacial conditions which lasted for ~ 8–12.5 kyr, and ends with glacial inception at ~ 774–777 ka. This inception has left an array of climatostratigraphic signals close to the Early–Middle Pleistocene boundary. MIS 19b–a contains a series of three or four interstadials often with rectangular-shaped waveforms and marked by abrupt (< 200 year) transitions. Intervening stadials including the inception of glaciation are linked to the calving of ice sheets into the northern North Atlantic and consequent disruption of the Atlantic meridional overturning circulation (AMOC), which by means of the thermal bipolar seesaw caused phase-lagged warming events in the Antarctic. The coherence of stadial–interstadial oscillations during MIS 19b–a across the Asian–Pacific and North Atlantic–Mediterranean realms suggests AMOC-originated shifts in the Intertropical Convergence Zone and pacing by equatorial insolation forcing. Low-latitude monsoon dynamics appear to have amplified responses regionally although high-latitude teleconnections may also have played a role.

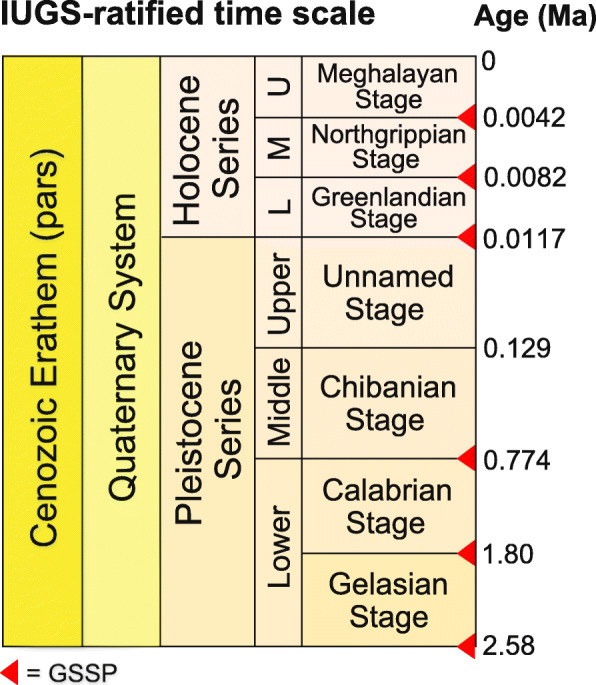

## Introduction

On January 17, 2020, the Executive Committee of the International Union of Geological Sciences ratified the Global Boundary Stratotype Section and Point (GSSP) defining the base of the Chibanian Stage and Middle Pleistocene Subseries at the Chiba section, Japan (Suganuma et al. in press), with an astronomically calibrated age of 774.1 ± 5.0 ka (Suganuma et al. 2018). This gave official recognition to the Middle Pleistocene, a term in use since the 1860s. The primary guide to this boundary is the Matuyama–Brunhes (M–B) paleomagnetic reversal which falls within Marine Isotope Stage (MIS) 19. Not only does MIS 19 allow the base of the Middle Pleistocene to be recognized independently of the M–B reversal and at millennial-scale resolution, but its earliest substage, MIS 19c, also serves as an orbital analogue for our own interglacial (e.g. Pol et al. 2010; Tzedakis et al. 2012a, 2012b; Yin and Berger 2015). This review examines the history behind the use of the term Middle Pleistocene, documents the procedure leading to the selection and ratification of the GSSP, examines and critiques the development of terminology used for MIS 19 and its subdivision, and synthesizes its climatic evolution on a global scale.

## The Middle Pleistocene and formal chronostratigraphy

**Fig. 1:**
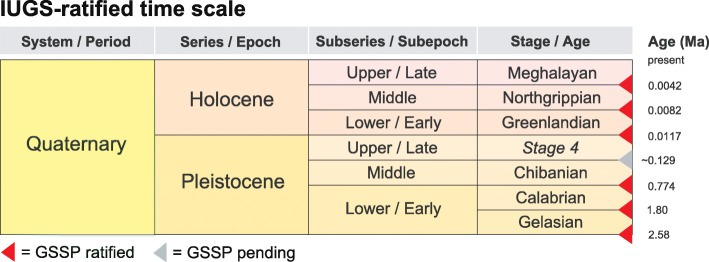
The Quaternary System/Period and its official subdivision as currently approved by the ICS and ratified by the IUGS EC. *Stage 4* corresponding to the Upper Pleistocene Subseries has yet to be officially defined. GSSP = Global Boundary Stratotype Section and Point (from Head et al. in press)

A GSSP is an internationally designated point within a stratotype. It serves as a global geostandard to define the base of an official unit (or coterminous units) within the International Chronostratigraphic Chart (Cohen et al. 2013 updated). This chart is administered by the International Commission on Stratigraphy (ICS), a constituent body of the International Union of Geological Sciences (IUGS), and it provides an officially approved framework for the geological time scale. The International Chronostratigraphic Chart is hierarchical in topology, with the base of each unit of higher rank defined by the base of the unit of next lower rank, a pattern that repeats down to the lowest unit definable by a GSSP, the stage (Salvador 1994; Remane et al. 1996). Accordingly, the Chiba GSSP defines both the Chibanian Stage and Middle Pleistocene Subseries (Fig. 1). A GSSP technically defines only the base of a chronostratigraphic unit, but in practice it marks the termination also of the top of the subjacent unit, in this case the Calabrian Stage and Lower Pleistocene Subseries. The top of the Chibanian Stage and Middle Pleistocene Subseries are presently not officially defined, except nominally by ratification of the term Upper Pleistocene Subseries which awaits official definition by a GSSP. The base of the Upper Pleistocene has a provisional age of ~ 129 ka (Head et al. in press).

**Fig. 2:**
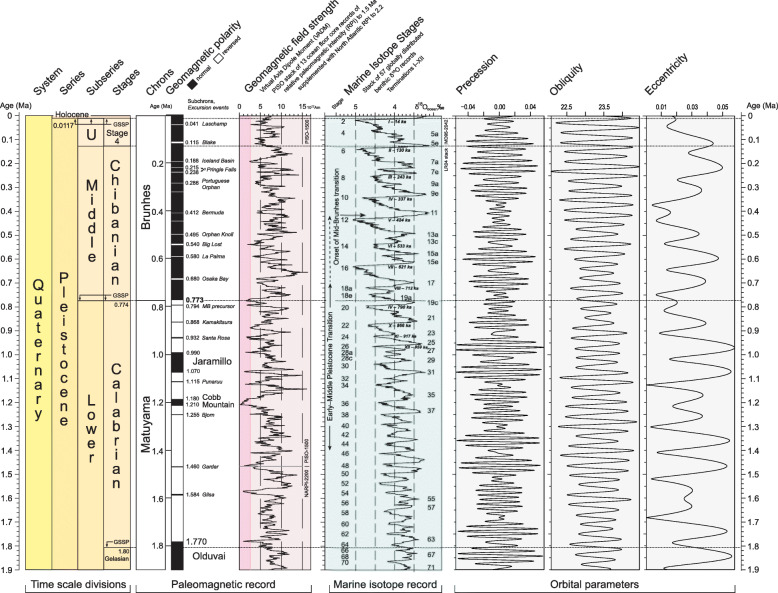
Stratigraphic correlation table and orbital parameters for the last 1.9 million years, including the Early–Middle Pleistocene transition (1.4–0.7 Ma, Sánchez-Goñi et al. 2019; or 1.4–0.4 Ma, Head and Gibbard 2015b). The time scale is based on Fig. 1; geomagnetic polarity reversals and field paleointensity data are from Cohen and Gibbard (2019) and Channell et al. (2016, 2020) with ages of reversals based on orbital tuning of the sedimentary record (Channell et al. 2020); marine isotope record and numbering of marine isotope stages is from Lisiecki and Raymo (2005), with ages of terminations from Lisiecki (undated) and selected substages from Railsback et al. (2015); orbital parameters representing precession (Laskar et al. 2004), obliquity (Laskar et al. 2004), and eccentricity (Laskar et al. 2011) are from Head and Gibbard (2015b). Updated from Cohen and Gibbard (2019)

It remains to be determined whether the Chibanian Stage will always precisely equate in extent with the Middle Pleistocene Subseries. There are good grounds for introducing a second stage for the Middle Pleistocene Subseries defined at its base by the onset of a major climatic event known as the “Mid-Brunhes Event” (Jansen et al. 1986) or “Mid-Brunhes Transition” (Yin 2013) which marks a step change in Quaternary climate. This climatic shift corresponds to an increase in the amplitude of quasi-100 kyr glacial–interglacial cycles and is marked by increases in interglacial sea-surface and Antarctic temperatures, atmospheric CO_2_, and CH_4_ levels, all beginning with Marine Isotope Stage (MIS) 11 (Barth et al. 2018). The onset of this transition is globally synchronous and corresponds to that between MIS 12 and MIS 11 (Termination V), dating to ~ 430 ka (Barth et al. 2018) (Fig. 2). It is readily identified in successions where astrochronology can be applied, including deep-ocean, ice-core, and European and Chinese loess records, and coincides with the base of Holsteinian Northwest European Stage, Likhvinian Russian Plain Stage and Zavadivian Ukrainian Loess Plain Stage (Cohen and Gibbard 2019). The Bermuda geomagnetic excursion, which lies at a prominent relative paleointensity minimum at ~ 412 ka in MIS 11c (Channell et al. 2020), could serve as an additional stratigraphic marker (Fig. 2). However, for now the Chibanian Stage extends upwards to the base of the Upper Pleistocene Subseries (Fig. 1).

The International Stratigraphic Guide distinguishes only between formal and informal stratigraphic terms. Formal terms “are properly defined and named according to an established or conventionally agreed scheme of classification ... The initial letter of the rank- or unit-term of named formal units is capitalized” (Salvador 1994, p. 14, see also p. 24). Those unit-terms appearing in the International Chronostratigraphic Chart are not merely formal terms but have also been approved by the ICS following extensive deliberation and then ratified by the Executive Committee of the IUGS (see Head 2019 for details of this process). These terms are here treated as “official” or “ratified” to distinguish them from formal terms lacking this approval (Head and Gibbard 2015a).

### History of the term Middle Pleistocene

**Fig. 3:**
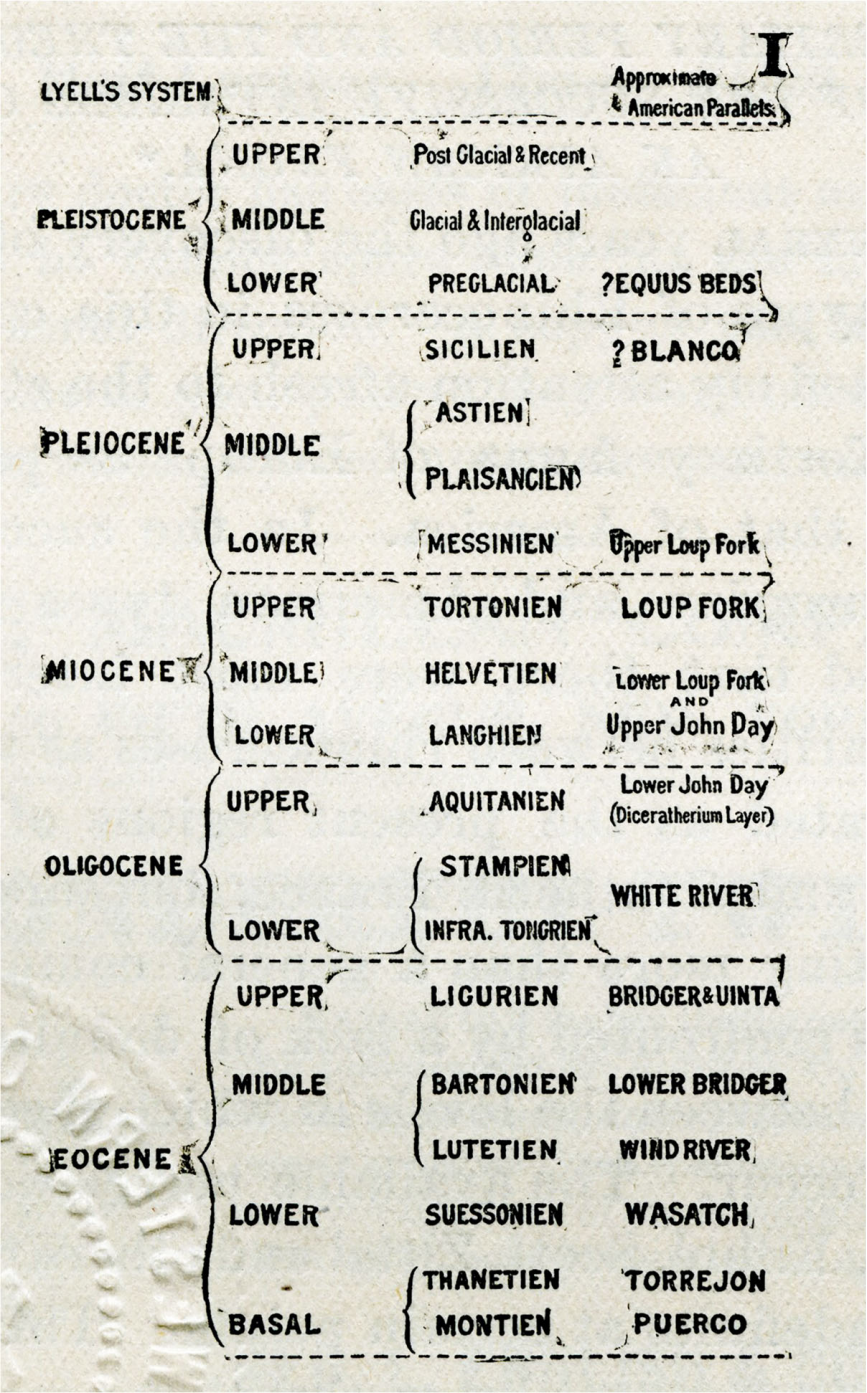
Reproduction of Chart 1 of Osborn (1900), an early example of the tripartite subdivision of the Pleistocene along with other Cenozoic series and their subdivision. The word Pleiocene, from the Greek *pleiōn* (Latinized as *plio-*), is a rare variant spelling of Pliocene

Charles Lyell in 1839 introduced the term Pleistocene (Greek, *pleīstos*, most; and *kainos*, recent) as a substitute for his Newer Pliocene (Lyell 1839, p. 621), but unlike his other series of the Cenozoic (Head et al. 2017), he refrained from dividing it into subseries. Indeed in 1863, Lyell proposed abandoning Pleistocene altogether on grounds that Forbes (1846) had popularized this term not in the sense of Lyell’s Newer Pliocene but almost precisely with reference to the subsequent interval of time for which Lyell was now introducing the term Post-pliocene (Lyell 1863, p. 6). By 1865, Lyell had conceded that if the term Pleistocene continued to be used, then it should not be as originally intended but in place of his “Post-pliocene” (Lyell 1865, footnote to p. 108). By the time Lyell had unconditionally accepted the Pleistocene in place of his Post-pliocene (Lyell 1873, p. 3, 4), this suggestion had already been generally adopted, with subdivision quickly following. The term “middle Pleistocene” for instance was employed informally by Harkness as early as 1869 (Harkness 1869), and the positional modifiers “early”, “middle”, and “late” have been used for the Pleistocene since at least the 1870s (e.g. Dawkins 1878). By 1900, this tripartite subdivision had become formalized in the English literature, with Osborn using the terms Lower Pleistocene (preglacial), Middle Pleistocene (glacial and interglacial, itself subdivided into lower, middle and upper), and Upper Pleistocene (postglacial and Recent) (Osborn 1900, p. 570, charts I and II) (Fig. 3).

This use of subseries for the Pleistocene had become entrenched by the time of the Second International Conference of the Association pour l’étude du Quaternaire européen (the forerunner of the International Union for Quaternary Research [INQUA] meetings) held in Leningrad in 1932, and subseries terms were used in a formal sense by Zeuner (1935, 1945) who in 1935 was already applying Milankovitch cyclicity and insolation curves to provide absolute dates for Pleistocene successions. In 1945, he considered the base of the Middle Pleistocene to have an age of ~ 425 ka.

The Japanese geophysicist Motonori Matsuyama (1884–1958, as spelled and pronounced but mistransliterated in his own publications and others as Matuyama) was the first to document clearly from basalts in the Genbudō (basalt caves), Japan (Matuyama 1929), the reversed magnetic polarity interval from 2.58 to 0.773 Ma that we now call the Matuyama Reversed Polarity Chron. However, it was the emergence of the geomagnetic polarity reversal time scale for the Pleistocene in the 1960s (Cox et al. 1963, 1964; Opdyke et al. 1966; Ninkovich et al. 1966; Glass et al. 1967; see Watkins 1972 for historical review), and particularly the recognition and radiometric dating of the M–B reversal and Jaramillo “event” (Doell and Dalrymple 1966), that created new possibilities for global stratigraphic correlation and Pleistocene time scale calibration. Accordingly, participants at the Burg Wartenstein Symposium “Stratigraphy and Patterns of Cultural Change in the Middle Pleistocene”, held in Austria in 1973, recommended that “The beginning of the Middle Pleistocene should be so defined as to either coincide with or be closely linked to the boundary between the Matuyama Reversed Epoch and the Brunhes Normal Epoch of paleomagnetic chronology” (Butzer and Isaac 1975, appendix 2, p. 901), as noted by Pillans (2003). In the same year, the INQUA Working Group on Major Subdivisions of the Pleistocene was established at the IX INQUA Congress in Christchurch, New Zealand, 1973, with its primary aim to define globally recognizable boundaries for the lower, middle, and upper Pleistocene subseries (Richmond 1996). The rank of subseries was adopted in preference to stage as the latter term was already used widely in Quaternary stratigraphy for locally and regionally defined units. At the XIIth INQUA Congress in Ottawa in 1987, the Working Group submitted a proposal, which was accepted by INQUA’s stratigraphic commission and approved by the congress, that “As evolutionary biostratigraphy is not able to provide boundaries that are as globally applicable and time-parallel as are possible by other means, the Lower–Middle Pleistocene boundary should be taken provisionally at the Matuyama–Brunhes palaeomagnetic reversal ...” (Anonymous 1988, p. 228; Richmond 1996, p. 320). From then on, the M–B reversal became the preferred and indeed de facto marker for the Early–Middle Pleistocene boundary (e.g. Bowen 1988; Berggren et al. 1995; Pillans 2003; Gradstein et al. 2005; Head and Gibbard 2005, 2015a, 2015b; Cita et al. 2006, 2008, Cita 2008; Head et al. 2008). Nonetheless, the Early–Middle Pleistocene boundary did not have official standing because this required the selection and approval of a GSSP.

### Selecting a primary guide for the base of the Middle Pleistocene Subseries

**Fig. 4:**
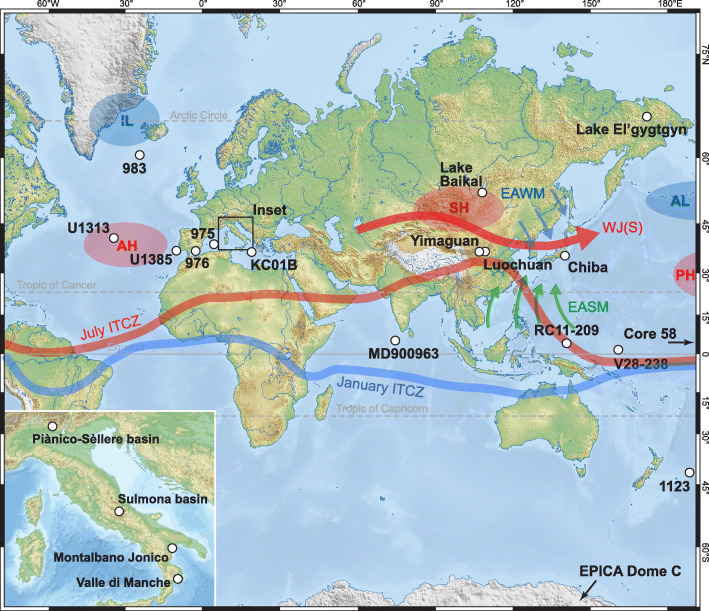
Location of sites discussed in the text and present atmospheric features. ODP Site 983 Gardar Drift, Iceland Basin; IODP Site U1313 upper western flank of the Mid-Atlantic Ridge, central North Atlantic; IODP Site U1385 southwest Portuguese margin; ODP Site 976 Alboran Sea; ODP Site 975 western Mediterranean Sea; Core KC01B Ionian Sea; Core MD900963 tropical Indian Ocean; Lake Baikal, SE Siberia; Yimaguan and Luochuan, Chinese Loess Plateau; Chiba composite section, Japan; Vema 28-238 and RC11-209 cores, western equatorial Pacific Ocean; ODP Site 1123 Chatham Ridge, South Pacific; EPICA Dome C ice core (75° 06′ S, 123° 21′ E, location is off the map); Core 58 of Arrhenius (1952), eastern equatorial Pacific Ocean (6° 44′ N, 129° 28′ W; location is off the map); inset shows important Italian sites. The Westerly Jet (WJ) during summer (S), East Asian Summer/Winter Monsoon (EASM/EAWM), and summer/winter variation in the position of the Intertropical Convergence Zone (ITCZ) adapted from Cheng et al. (2012) and Liu et al. (2015); the Siberian High (SH) and Aleutian Low (AL) are primarily winter atmospheric pressure systems; the AL and Pacific High (PH) form the North Pacific Oscillation; the Islandic Low (IL) and Azores High (AH) form the North Atlantic Oscillation

The XIVth INQUA Congress in Berlin in 1995 focused on three potential candidate GSSPs: Chiba in Japan, Montalbano Jonico in Basilicata, Italy, and the Wanganui Basin in New Zealand (Pillans 2003), the latter being discounted because it contained unconformities (Head et al. 2008). Meanwhile, the ICS Subcommission on Quaternary Stratigraphy (SQS), in 2002 after a period of inactivity, established a Working Group to review all aspects of the Early–Middle Pleistocene boundary including the selection of a suitable GSSP (Head et al. 2008). At the 32nd International Geological Congress in Florence in 2004, the Early–Middle Pleistocene boundary Working Group recommended that (1) The boundary be defined in a marine section at a point “close to” the Matuyama–Brunhes palaeomagnetic reversal, where the definition of “close” was agreed to mean within plus or minus one isotope stage of the reversal; and (2) the GSSP should be located in a marine section exposed on land, not in a deep sea core (Head et al. 2008). A third potential candidate GSSP emerged at the Florence congress: the Valle di Manche section in Calabria, Italy (Capraro et al. 2004, 2005) (Fig. 4).

Deciding upon the primary guide to the boundary should be made prior to the consideration of candidate sections because the expression of this guide in the GSSP must be exemplary (Remane et al. 1996). The Working Group’s decisions at Florence were therefore crucial in moving the process forward. The M–B reversal with an age of ~ 773 ka (Singer et al. 2019; Channell et al. 2020; Haneda et al. 2020a; and earlier reviews by Head and Gibbard 2005, 2015b) was chosen in part because it (1) has an isochronous expression in most marine and terrestrial sediments and even in ice cores, (2) is the most prominent geomagnetic field reversal in the past 773 kyr, and (3) occurs within the Early–Middle Pleistocene transition (1.4–0.7 or 1.4–0.4 Ma; Fig. 2), aligning the Early–Middle Pleistocene boundary with a fundamental shift in Earth’s history. This shift from a 41 ky to quasi-100 ky orbital rhythm was marked by increases in the amplitude of climate oscillations and in long-term average global ice volume, and by strong asymmetry in global ice volume cycles. It resulted in progressive and fundamental physical, chemical, climatic, and biotic adjustments to the planet (Head and Gibbard 2015b).

### Voting on candidates for the Middle Pleistocene Subseries GSSP

The three final candidates for the Early–Middle Pleistocene GSSP were the Valle di Manche section in Calabria and the Ideale section at Montalbano Jonico in Basilicata, both in Italy, and the Chiba section in Japan (Head and Gibbard 2015a) (Fig. 4). Following field trips that allowed members of the Working Group to visit all three sites in advance of voting (Ciaranfi et al. 2015; Okada and Suganuma 2018), the vigorous and exhaustive process of selecting a GSSP began on July 11, 2017, with the circulation of proposals to the membership of the Working Group (Table 1). It had been decided by all three proponents in advance that the proposals should remain confidential because they contained unpublished material. This confidentiality was respected through the entire selection process. Discussions started on July 25 and ended at the close of October 3, 2017, allowing an extended opportunity to exchange views. Discussions were wide-ranging, in acknowledgement that a GSSP must record an array of stratigraphic markers, but inevitably focused on the M–B reversal. A detailed commentary on these discussions is given in Head (2019) and only key aspects will be presented here.

**Table 1:** Members of the SQS Working Group on the Early–Middle Pleistocene Boundary in 2017 at the time of voting on the GSSP

• Luca Capraro, Dipartimento di Geoscienze, Università degli Studi di Padova, Padova, Italy	
• Bradford M. Clement, Integrated Ocean Drilling Program and Department of Geology and Geophysics, Texas A&M University, College Station, USA	
• Mauro Coltorti, Dipartimento di Scienze Fisiche, della Terra e dell’Ambiente, Università di Siena, Siena, Italy	
• Craig S. Feibel, Department of Earth and Planetary Sciences, Rutgers University, Piscataway, New Jersey, USA	
• Martin J. Head, Department of Earth Sciences, Brock University, St. Catharines, Ontario, Canada (Co-Convener)	
• Lorraine E. Lisiecki, Department of Earth Science, University of California, Santa Barbara, USA	
• Jiaqi Liu, Institute of Geology and Geophysics, Chinese Academy of Sciences, Beijing, China	
• Maria Marino, Dipartimento di Scienze della Terra e Geoambientali, Università degli Studi di Bari Aldo Moro, Bari, Italy	
• Anastasia K. Markova, Institute of Geography, Russian Academy of Sciences, Moscow, Russia	
• Brad Pillans, Research School of Earth Sciences, Australian National University, Canberra, ACT, Australia (Co-Convener)	
• Yoshiki Saito, Geological Survey of Japan, AIST, Tsukuba, Ibaraki, Japan; and Estuary Research Center, Shimane University, Matsue, Shimane, Japan	
• Brad S. Singer, Department of Geoscience, University of Wisconsin–Madison, USA	
• Yusuke Suganuma, National Institute of Polar Research, Tachikawa, Tokyo, Japan and Department of Polar Science, School of Multidisciplinary Sciences, The Graduate University for Advanced Studies (SOKENDAI), Tachikawa, Tokyo, Japan	
• Charles Turner, Department of Earth Sciences, The Open University, Milton Keynes, UK	
• Chronis Tzedakis, Department of Geography, University College London, London, UK	
• Thijs van Kolfschoten, Faculty of Archaeology, Leiden University, Leiden, The Netherlands	

The M–B reversal in the Chiba composite section (CbCS) is expressed by directional changes (virtual geomagnetic pole [VGP] latitudes) and changes in the geomagnetic field intensity based on both the paleomagnetic record and a coherent record of its proxy, the authigenic ^10^Be/^9^Be record (Suganuma et al. 2015; Okada et al. 2017; Simon et al. 2019; Haneda et al. 2020a). These studies are based on an astronomical age model introduced by Suganuma et al. (2015) and refined by Okada et al. (2017) and again by Suganuma et al. (2018). Okada et al. (2017) determined the directional midpoint at 771.7 ka with a duration of 2.8 kyr; these values revised to 772.9 ka and 1.9 kyr on the age model of Suganuma et al. (2018). Simon et al. (2019) using new paleomagnetic data reported a directional switch between 773.9 and 771.9 ka, with a duration therefore of 2.0 kyr. Haneda et al. (2020a) using new paleomagnetic data combined with earlier studies (Suganuma et al. 2015; Hyodo et al. 2016; Okada et al. 2017) determined the average directional midpoint at 772.9 ka with a duration of 1.1 kyr based on the age model of Suganuma et al. (2018). Allowing for a 5 kyr chronological uncertainty in the orbital tuning of the CbCS (4 kyr from Lisiecki and Raymo 2005, and 1 kyr from Elderfield et al. 2012; see Suganuma et al. in press) and a stratigraphic uncertainty of 0.4 ka (Haneda et al., 2020a), the astronomical age of the directional midpoint of the M–B reversal is 772.9 ± 5.4 ka, with a duration of up to ~ 2 kyr. The close match between the geomagnetic field intensity and the ^10^Be/^9^Be record confirms that any lock-in depth offset (Roberts and Winklhofer 2004; Suganuma et al. 2010, 2011) at this high sedimentation rate site (89 cm/kyr across the boundary) is minimal. This age closely accords with ages of around 773 ka from other well constrained sites (Channell 2017; Channell et al. 2020; Singer et al. 2019; Valet et al. 2019; Haneda et al. 2020a; earlier records reviewed in Head and Gibbard 2015b). The geomagnetic field intensity record shows two pronounced minima, one at 772 ka near the polarity switch and the other at 764 ka (Simon et al. 2019). It is therefore evident that the position of the VGP switch cannot be precisely predicted using geomagnetic field intensity data alone.

Montalbano Jonico lacks a paleomagnetic record owing to late diagenetic remagnetization associated with the growth of greigite (Sagnotti et al. 2010). A ^10^Be/^9^Be record at this site serves as a proxy for the geomagnetic field intensity site and reveals a peak (field intensity minimum) at the approximate position of the M–B reversal as determined by the marine isotope record (Simon et al. 2017; Nomade et al. 2019) and dated by an ^40^Ar/^39^Ar age of 774.1 ± 0.9 ka for tephra layer V4 which coincides with the ^10^Be/^9^Be peak (Nomade et al. 2019). While this corroborates the position and age of the M–B reversal in this part of the Mediterranean, the geomagnetic field intensity alone is insufficient to identify the *precise* position of the polarity switch (see Channell et al. 2020), as demonstrated for Chiba and elsewhere.

The M–B reversal as recorded at the Valle di Manche (Capraro et al. 2017) has been astronomically dated at 786.9 ± 5 ka (Macrì et al. 2018), an anomalously old age when compared with most global records including the ^10^Be/^9^Be proxy record of Montalbano Jonico section just 135 km to the north (Head 2019). A ^10^Be/^9^Be record at the Valle di Manche section gives a peak in ^10^Be concentration ∼3.5 m above the reported M–B reversal. This translates to a difference of ∼12 kyr (Capraro et al. 2018) and is coincident with the age of this reversal elsewhere. Lock-in depth seems unable to explain the spuriously low position of the reversal because sedimentation rates at ∼27 cm/kyr in this part of the Valle di Manche section are reasonably high (Macrì et al. 2018). When the ^10^Be/^9^Be curves for the Valle di Manche and Montalbano Jonico sections are compared, they show strong agreement (Capraro et al. 2019). The ^10^Be/^9^Be peak therefore most likely marks the true position of the M–B Chron boundary at both sections, with the Valle di Manche paleomagnetic reversal ∼3.5 m below representing diagenetic overprinting and remagnetization (Head 2019; but see Capraro et al. 2019 for an alternative interpretation). This explanation would also account for the unusually rapid directional transition of this reversal in the order of 100 years or less at the Valle di Manche section (Macrì et al. 2018). A similar relatively old (786.1 ± 1.5 ka) M–B reversal, perhaps with an even more rapid transition, reported from the Sulmona basin in central Italy (Sagnotti et al. 2014; Sagnotti et al. 2016) has been restudied and appears to carry an unreliable signal (Evans and Muxworthy 2018; but see Sagnotti et al. 2018). Another relatively old age (~ 779 ka) for the reversal has been reported from Site IODP U1385 off Portugal (Sánchez-Goñi et al. 2016). The position of this reversal has since been revised, and it is now provisionally placed higher in the core than reported from shipboard analysis (Xuan Chuang, pers. comm. 2018). Moreover, a reported M–B reversal age of 783.4 ± 0.6 ka at ODP Site 758 in the Indian Ocean (Mark et al. 2017) has been challenged on grounds that the sedimentation rates and hence resolution of the isotope and magnetic stratigraphies are all too low for precise age determination (Channell and Hodell 2017).

It had been decided in advance that the choice individual members made when voting within the Working Group would not be revealed, contrary to usual practice within ICS. Because of active and potential research collaborations within the group, to do otherwise might have compromised the vote. Voting by the SQS Working Group commenced on October 10, 2017, and concluded on November 10, 2017. As noted in Head (2019), the Chiba proposal was passed by supermajority, gaining 73% of the total votes cast.

### Final approval and ratification of the Chiba GSSP

Following minor revision, the Chiba proposal was submitted to the SQS voting membership for discussion and voting, this process concluding on 16 November 2018 with a supermajority of 86% in favour of the Chiba proposal. Discussion within the ICS voting membership began on August 16, 2019, and closed on October 28, 2019. Voting concluded on November 28, 2019, with the results as follows: 17 in favour, 2 against, no abstentions, all ballots returned. The proposal was therefore carried with a supermajority of 89.5%. This ICS-approved proposal for the Chibanian Stage/Age and Middle Pleistocene Subseries/Subepoch was ratified in full by the IUGS EC on January 17, 2020, drawing to a close a process initiated by INQUA in 1973, some 47 years earlier.

The GSSP is placed at the base of a regional lithostratigraphic marker, the Ontake-Byakubi-E (Byk-E) tephra bed (Takeshita et al. 2016), in the Chiba section. It has an astronomical age of 774.1 ka (Suganuma et al. in press) and a zircon U-Pb age of 772.7 ± 7.2 ka (Suganuma et al. 2015), occurring immediately below the top of Marine Isotope Substage 19c. The directional midpoint of the M–B reversal, serving as the primary guide to the boundary, is just 1.1 m above the GSSP and has an astronomical age of 772.9 ± 5.4  ka (Haneda et al. 2020a; Suganuma et al. in press). The numerous climatostratigraphic signals associated with the MIS 19c/b transition, which represents the inception of glaciation for MIS 19 (see below), provide additional means to identify this boundary precisely on a global scale.

IUGS ratification of the Middle Pleistocene Subseries officially legitimized a unit-rank term already in wide and formal use within the Quaternary community (Head et al. 2017), and the ratification of an accompanying stage complied with the requirements of the International Commission on Stratigraphy. INQUA fully supported ratification of both stage and subseries (van Kolfschoten 2020). This also provided Japan with its first GSSP, coincidentally based on a paleomagnetic reversal first clearly documented in Japan by Motonori Matsuyama, an early Japanese pioneer of magnetostratigraphy. The achievements of Japanese geophysicist Naoto Kawai may also be recalled, as he was the first to record a paleomagnetic reversal in sedimentary rocks (Kawai 1951).

## Marine Isotope Stage 19

MIS 19 has long been associated with the M–B reversal, and this interglacial stage therefore provides a well-documented cluster of additional stratigraphic signals to identify the base of the Chibanian Stage on a global scale. Its climatic evolution is also significant because MIS 19c serves as an orbital analogue for the present interglacial (e.g. Berger and Loutre 1991; Pol et al. 2010; Tzedakis et al. 2012a, 2012b; Yin and Berger 2015) and therefore provides a natural baseline for assessing our future climate.

### History of MIS 19

**Fig. 5:**
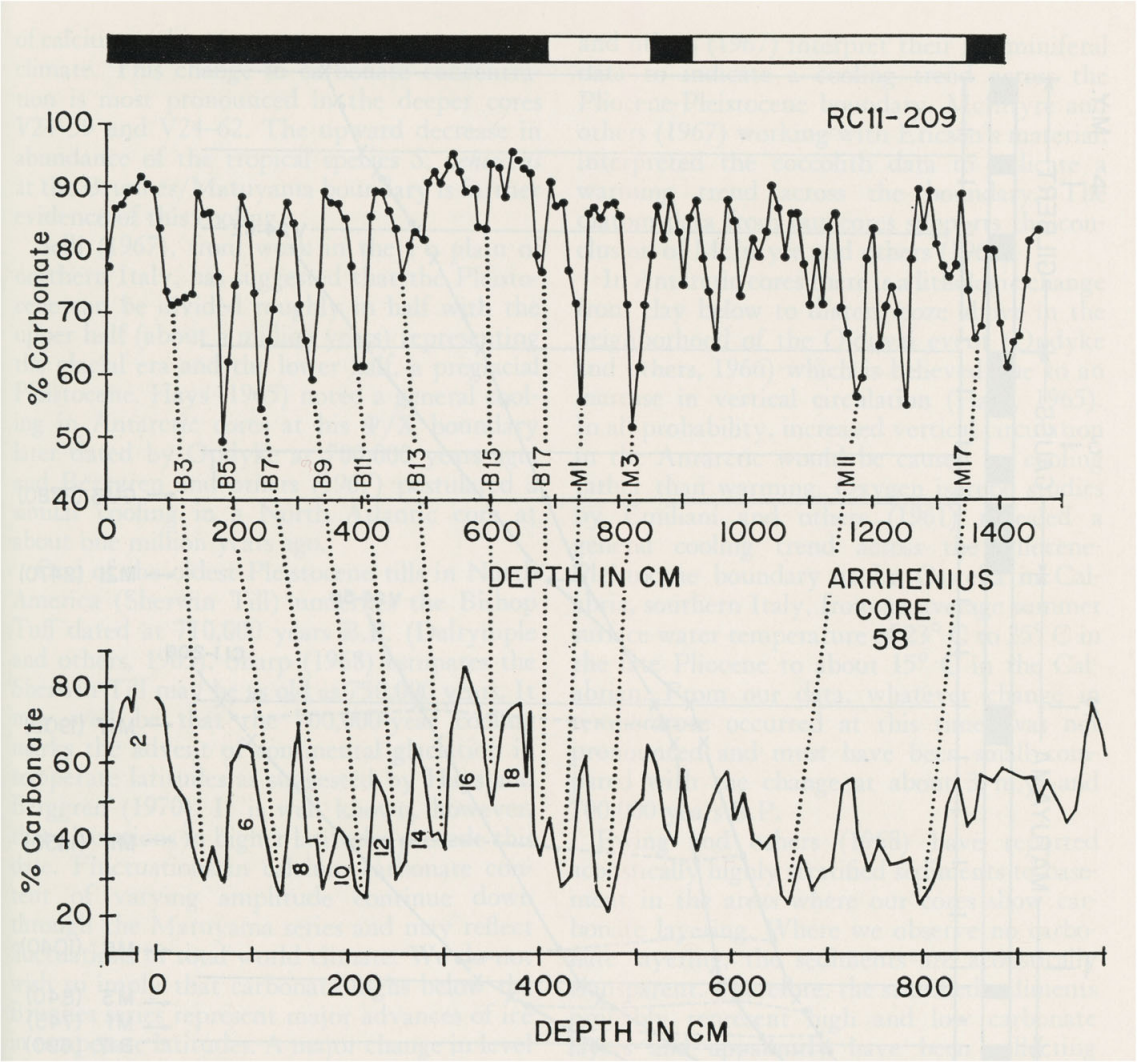
Reproduction of fig. 14 in Hays et al. (1969) showing correlation between carbonate percentage in equatorial Pacific core RC11-209 and that of east equatorial Pacific core 58 of Arrhenius (1952). Carbonate cycle B17 in core RC11-209 corresponds to an unlabelled cycle in Arrhenius’ core 58. This would have been cycle 19 had Arrhenius continued labelling. Cycle B17 aligns with the Matuyama–Brunhes paleomagnetic reversal and represents MIS 19

**Fig. 6:**
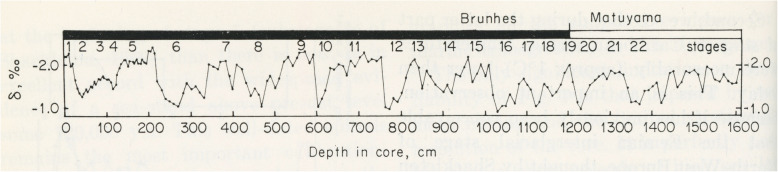
Reproduction of fig. 9 in Shackleton and Opdyke (1973) showing the δ^18^O record of the planktonic foraminifera *Globigerinoides sacculifer*a from core V28-238, western equatorial Pacific, from which MIS 19 was defined for the first time (Shackleton and Opdyke 1973). This study confirms the links between MIS 19, carbonate cycle B17 of Hays et al. (1969), and the Matuyama–Brunhes paleomagnetic reversal

In labelling fluctuating percentages of carbonate in marine cores from the equatorial Pacific Ocean, Arrhenius (1952) introduced a numbering system in which even/odd numbers represent glacial/interglacial cycles. Arrhenius correctly surmised that carbonate-rich layers represent increased productivity linked to upwelling driven by strengthened trade winds during glacial intervals. Arrhenius labelled 18 carbonate cycles, recording although not labelling older cycles including the equivalent of what was to be known as MIS 19 (Fig. 5). Hays et al. (1969) continued this research through additional cores in the Pacific. They labelled as B17 (where B = Brunhes) a carbonate-poor interglacial cycle coinciding with the base of the Brunhes Chron (Fig. 5). Emiliani’s (1955, 1966) original oxygen isotope stages followed the numbering scheme of Arrhenius. Shackleton and Opdyke (1973) in their now famous oxygen isotope and magnetostratigraphic analysis of the Vema 28-238 core from the western equatorial Pacific Ocean (V28-238 in Fig. 4) extended to Stage 22 Emiliani’s original oxygen isotope stages 1–14 (Emiliani 1955) and then 1–17 (Emiliani 1966). In doing so, Shackleton and Opdyke (1973) were the first to label MIS 19 (Fig. 6). They equated cycle B17 of Hays et al. (1969) with their MIS 19, confirming the association of this interglacial stage with the M–B reversal.

### Subdivision of MIS 19

The division of marine isotope stages into substages has a long history beginning with Shackleton (1969) who subdivided MIS 5 into five lettered substages, a–e (Railsback et al. 2015). As noted by Railsback et al. (2015), a parallel system of subdividing marine isotope stages into decimal-style numbered “events” has its roots in the labelling system of Arrhenius (1952) and was first applied to marine isotope stages by Prell et al. (1986; but see Railsback et al. 2015 for historical development) who reasoned that defining events (peaks and troughs) rather than stages (intervals of sediment or time) was more useful in applying tie points for age models. Although the two approaches tended to be used rather indiscriminately and interchangeably, Shackleton (2006) remarked that conceptually they are different and not interchangeable. He noted that “events” relied upon peak values in analyses that are more difficult to replicate in practice, and hence reliably correlate, than the midpoints of transitions that define substage boundaries. This midpoint approach is indeed is how stages themselves are defined following Emiliani (1955). Accordingly, Shackleton (2006), Railsback et al. (2015) in their extensive review, and the present study, have all favoured contiguous lettered subdivisions for marine isotope stages.

#### Subdivision used in the present study

**Fig. 7:**
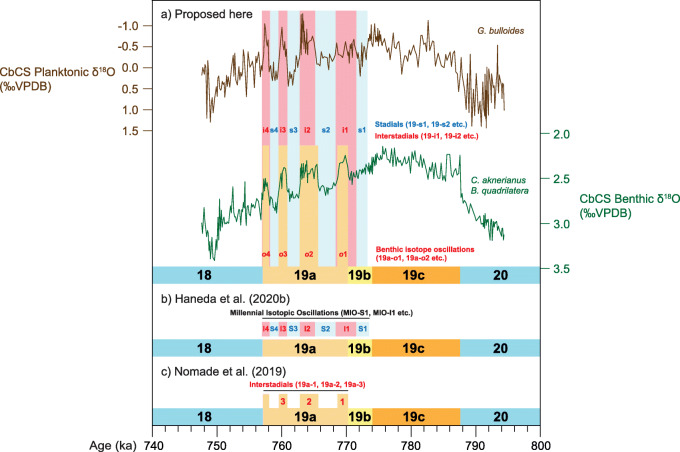
Differing subdivisions of the interval comprising Marine Isotope Substages 19b and 19a illustrated using the isotope record of the Chiba composite section (CbCS; Haneda et al. 2020b). (**a**) Four millennial-scale benthic isotope oscillations (MIS 19a-*o*1 to MIS 19a-*o*4) represented in the benthic foraminiferal record by lighter values exclusively within MIS 19a. Four stadials (MIS 19-s1 to MIS 19-s4) and four interstadials (MIS 19-i1 to MIS 19-i4) reflect more localized millennial-scale paleoenvironmental alternations across MIS 19b–a, in this case characterized by the planktonic foraminiferal record. (**b**) The labelling scheme of Haneda et al. (2020b) based on the planktonic foraminiferal record of the CbCS. (**c**) The labelling scheme of Nomade et al. (2019) based on the benthic foraminiferal record of Montalbano Jonico, Italy

The scheme used here is illustrated by its application to the CbCS record (Fig. 7). Three substages, 19c, 19b, and 19a, are recognized. MIS 19c comprises full interglacial conditions together with the rise to lighter foraminiferal δ^18^O values at the beginning of MIS 19 (Termination IX) and the decline to heavier values towards the end of MIS 19c, terminating with a glacial inception (Tzedakis et al. 2012a, 2012b). MIS 19b represents a single interval of heavier foraminiferal isotopic values (Nomade et al. 2019) which is recognized at the CbCS within the benthic record (Haneda et al. 2020b). The benthic foraminiferal δ^18^O record of MIS 19a is represented by a series of millennial-scale oscillations, with as many as four peaks of lighter isotopic values here labelled as MIS 19a-*o*1 to MIS 19a-*o*4, where “*o*” refers to *benthic isotope oscillation*. MIS 19a begins with MIS 19a-*o*1 (Fig. 7).

Superimposed on this benthic foraminiferal isotopic record through MIS 19b–a is as many as four stadial–interstadial alternations, here labelled MIS 19-s1 to MIS 19-s4 (stadials) and MIS 19-i1 to MIS 19-i4 (interstadials). MIS 19-s1 is the first of these millennial-scale climatic episodes and broadly coincides with the glacial inception marked by MIS 19b. They are recognized primarily in planktonic records including planktonic foraminiferal δ^18^O, but may be observed in pollen spectra and other terrestrial proxies.

Figure 7 shows how the labelling scheme presented here differs from those of Nomade et al. (2019) and Haneda et al. (2020b) as applied to the isotopic record of the CbCS. The present scheme does not preclude the use of additional biozones and informal event stratigraphy through all or part of MIS 19 where such detail is needed. The rationale for this subdivision is discussed in Section 3.2.3.

#### Division into substages

**Fig. 8:**
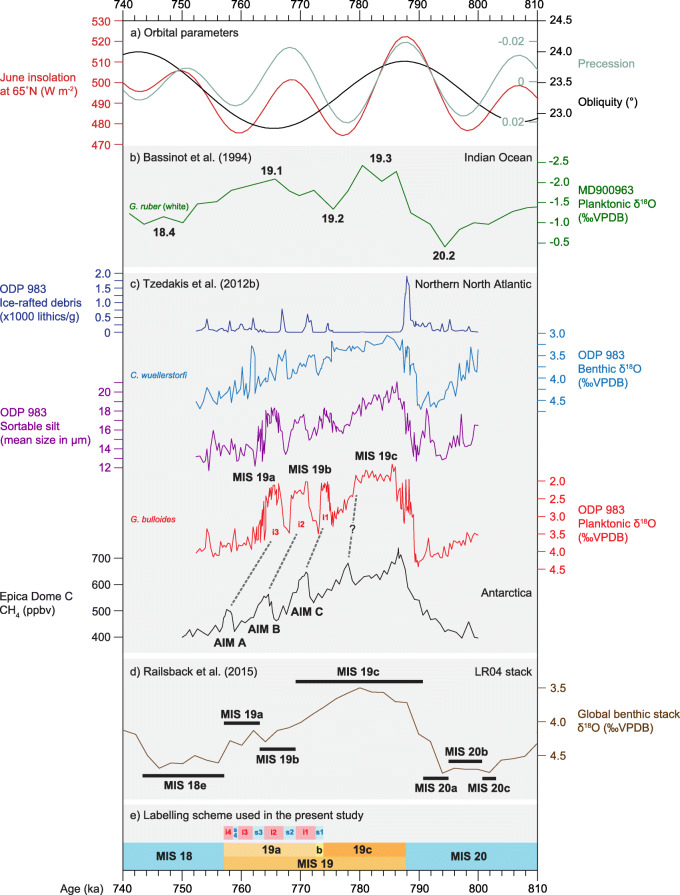
North Atlantic and global records of Marine Isotope Stage 19 (see also Fig. 9). (**a**) Insolation at 65° N in June, and precession and obliquity parameters. (**b**) Designation of events by Bassinot et al. (1994) based on the planktonic foraminiferal δ^18^O record of Indian Ocean core MD900963. (**c**) Lettered substages of Tzedakis et al. (2012b) applied to the foraminiferal δ^18^O and other records of ODP Site 983 Iceland Basin, with peaks correlated to Antarctic Isotope Maxima (AIMs) reflected in the Antarctic ice-core methane record from EPICA Dome C. Also included are the ice-rafted debris and sortable silt mean size (mean of 10–63 μm fraction) records (Kleiven et al. 2011). (**d**) Lettered substages of Railsback et al. (2015) as applied to the LR04 benthic δ^18^O foraminiferal stack of Lisiecki and Raymo (2005). (**e**) The MIS 19 subdivisional scheme used here (Fig. 7): interstadials i1, i2, and i3 are labelled in red. All records are plotted on their own published time scales and use the original substage designations

Bassinot et al. (1994) were the first to subdivide MIS 19 formally, defining MIS 19.1, 19.2, and 19.3 (Fig. 8b) on the basis of two pronounced planktonic foraminiferal δ^18^O peaks recorded from Core MD900963 in the tropical Indian Ocean (Fig. 4). No explanation was given for these two peaks although precession is strongly expressed in this core.

Tzedakis et al. (2012a, 2012b) seem to have initiated the application of lettered substages for MIS 19, with Tzedakis et al. (2012b, their fig. 4) applying MIS 19a, 19b, and 19c to the foraminiferal δ^18^O and ice-rafted debris (IRD) record from ODP Site 983 on the Gardar Drift, North Atlantic (Figs. 4 and 8c) and correlating this to the Antarctic ice-core record of EPICA Dome C (Figs. 4 and 8c). Tzedakis et al. (2012b) did not define the boundaries of their substages but MIS 19c clearly represents the rise to lightest isotopic values and the ensuing peak or plateau followed by a gradual decline to heavier values in the upper part of MIS 19c. MIS 19b and MIS 19a together represent three succeeding peaks of light isotopic values, here labelled interstadials i1, i2, and i3, that characterize the upper part of MIS 19. MIS 19b includes the two lowest interstadials and MIS 19a includes the third. Tzedakis et al. (2012b) correlated these three interstadials in the upper part of MIS 19 at Site 983 with three Antarctic Isotope Maxima (AIMs) documented in the EPICA Dome C ice-core record (EPICA Community Members 2006). Hence, the two lowest interstadials, assigned to MIS 19b, were correlated to AIM C and B, and the uppermost interstadial, assigned to MIS 19a, was correlated to AIM A (Fig. 8c).

Railsback et al. (2015) similarly subdivided MIS 19 into substages a, b, and c, but defined them with respect to the LR04 global benthic foraminiferal δ^18^O stack of Lisiecki and Raymo (2005) which only clearly distinguishes two peaks in the upper part of MIS 19. Railsback et al. (2015) assigned both peaks to MIS 19a and the preceding trough to MIS 19b (Fig. 8d). This scheme therefore differed significantly from that of Tzedakis et al. (2012b).

**Fig. 9:**
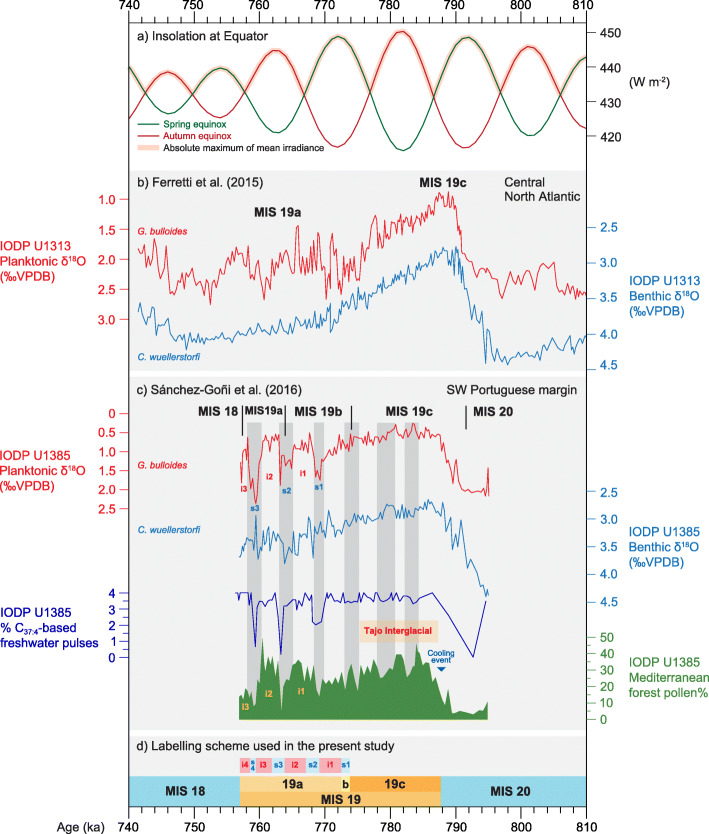
North Atlantic records of Marine Isotope Stage 19 (see also Fig. 8). (**a**) Insolation at Equator in spring and autumn, and absolute maximum of mean irradiance (Laskar et al. 2004; Ferretti et al. 2015; Haneda et al. 2020b). (**b**) IODP Site U1313 central North Atlantic: foraminiferal δ^18^O (Ferretti et al. 2015). (**c**) IODP Site U1385 southwest Portuguese margin: foraminiferal δ^18^O, alkenones (C37:4), and pollen records with grey bars indicating major contractions of the Mediterranean forest; the Tajo Interglacial occurs within MIS 19c, and a dark blue triangle marks a brief cooling event just before it (Sánchez-Goñi et al. 2016); stadials (s1–s3) and interstadials (i1–i3) are labelled following the correlations of Regattieri et al. (2019) and Nomade et al. (2019). (**d**) The MIS 19 subdivisional scheme used here (Fig. 7). All records are plotted on their own published time scales and use the original substage designations

Ferretti et al. (2015) published detailed benthic and planktonic foraminiferal δ^18^O records from IODP Site U1313 in the central North Atlantic (Fig. 4), although the upper part of MIS 19 could not be unambiguously resolved into three interstadials. MIS 19c and MIS 19a were therefore labelled only approximately and MIS 19b was omitted (Fig. 9b).

Sánchez-Goñi et al. (2016) in their study of IODP Site U1385 off southwest Portugal (Fig. 4) extended the upper boundary of MIS 19c to the top of the plateau of lightest δ^18^O values. The progressive decline to heavier values as well as the first of three conspicuous peaks in the upper part of MIS 19 were assigned to MIS 19b. The second and third peaks were assigned to MIS 19a (Fig. 9c). This scheme essentially follows that of Tzedakis et al. (2012b). Sánchez-Goñi et al. (2016) determined the MIS 20/19 and MIS 19/18 boundaries at the midpoints between highest/lowest and lowest/highest values in the δ^18^O benthic foraminiferal record, an approach following that of Shackleton et al. (2003) for establishing the MIS 6/5e and MIS 5e/d boundaries. They then applied the same method to determine the positions of the MIS 19c/b and MIS 19b/a boundaries, finding that the midpoints were broadly similar to positions they had statistically identified by the “Change point method” of Zeileis et al. (2002, 2003). Therefore, these limits and the substage classification they embody may reflect significant changes in global ice volume (Sánchez-Goñi et al. 2016). This method does not appear to have been used on other foraminiferal benthic records of MIS 19 to test such a possibility, but the shape of the δ^18^O benthic foraminiferal record at the CbCS in Japan (Fig. 7), for example, is rather different from that at Site U1385 especially across the MIS 19b–a interval.

**Fig. 10:**
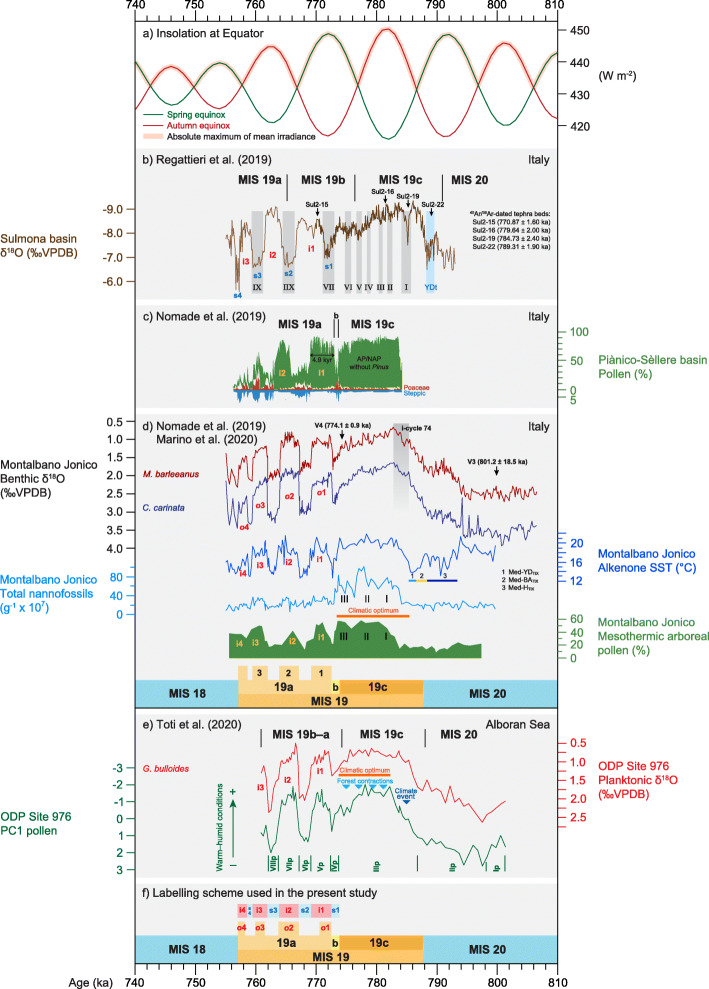
Mediterranean records of Marine Isotope Stage 19. (**a**) Insolation at Equator in spring and autumn, and absolute maximum of mean irradiance (Laskar et al. 2004; Ferretti et al. 2015; Haneda et al. 2020b). (**b**) Lacustrine Sulmona basin, central Italy: endogenic calcite δ^18^O, events I–IX of reduced precipitation based on δ^18^O, and tephra beds (positions based on modelled ages with ^40^Ar/^39^Ar ages shown separately) used to construct the age model, all from Regattieri et al. (2019); Younger Dryas-like event (YDt) from Giaccio et al. (2015). (**c**) Lacustrine Piànico-Sèllere basin, northern Italy: pollen (Nomade et al. 2019). (**d**) Ideale section, Montalbano Jonico, Italy: benthic foraminiferal δ^18^O and supporting age control using ^40^Ar/^39^Ar-dated tephra (volcaniclastic) beds V3 and V4 from Nomade et al. (2019). Ghost sapropel assigned to insolation cycle 74, alkenone sea-surface temperature (SST) with Heinrich-type (Med-H_TIX_) Mediterranean Bølling-Allerød-type (Med-BA_TIX_), and Younger-Dryas-type (Med-YD_TIX_) episodes associated with Termination IX, and total coccolith abundance and mesothermic arboreal pollen records showing phases I–III of climatic amelioration; from Marino et al. (2020). (**e**) ODP Site 976 Alboran Sea, western Mediterranean: planktonic foraminiferal δ^18^O and pollen (first component of Principal Component Analysis); light blue triangles show temperate forest contractions within MIS 19c and correspond to increases in total coccolithophore abundance; dark blue triangle represents a climate event marked by abundant Asteraceae pollen (Toti et al. 2020). The subdivisional scheme of Nomade et al. (2019) is given. (**f**) The MIS 19 subdivisional scheme used here (Fig. 7): benthic marine isotope oscillations *o*1–*o*4 are labelled in red, interstadials i1–i4 are labelled in red and orange. All records are plotted on their own published time scales and use the original substage designations

Regattieri et al. (2019) in their study of the lacustrine Sulmona basin in central Italy (Figs. 4 and 10b) broadly followed the lettered substage classification of Sánchez-Goñi et al. (2016). Their MIS 19b, the base of which is placed between two reduced-precipitation events (V and VI), includes stadial s1, interstadial i1 and part of the following stadial, s2. The MIS 19b–a boundary is drawn midway through their event IIX, here labelled stadial s2. In total, three interstadials are recognized within the MIS 19b–a interval at Sulmona (Fig. 10b), as with IODP Site U1385.

The lacustrine Piànico-Sèllere basin of northern Italy (Fig. 4) contains a finely resolved pollen record established by Moscariello et al. (2000). Although previously assigned to MIS 11, it most likely represents MIS 19 based on tephrochronology (Pinti et al. 2001, 2007; Roulleau et al. 2009) and paleoclimatic concordance with other Italian sites (Nomade et al. 2019). Nomade et al. (2019) labelled the Piànico-Sèllere pollen record (Fig. 10c) following their new classification of the Ideale section of Montalbano Jonico which recognizes at least three interstadials for MIS 19a and restricts MIS 19b to a single stadial, here labelled s1. Although based on pollen percentages, the record of the Piànico-Sèllere basin allows precise comparison with the marine isotope substages and recognition of at least interstadials i1 and i2 in MIS 19a.

The Ideale section of Montalbano Jonico, Basilicata, Italy (Fig. 4), yields one of the most detailed δ^18^O records of MIS 19 available (Simon et al. 2017; Nomade et al. 2019). MIS 19b is restricted to a brief cooling event following MIS 19c, and MIS 19a includes discrete intervals of lighter isotopic values labelled by Nomade et al. (2019) as interstadials 1 through 3, with a fourth labelled in the present study (fig. 8b in Nomade et al. 2019; Fig. 10d).

A somewhat truncated planktonic foraminiferal δ^18^O record of ODP Site 976, Alboran Sea, western Mediterranean (Toti et al. 2020; Fig. 4) does not distinguish between MIS 19b and MIS 19a, although two interstadials and the onset of a third in the upper part of MIS 19 can be referred to interstadials i1–i3 (Fig. 10e).

**Fig. 11:**
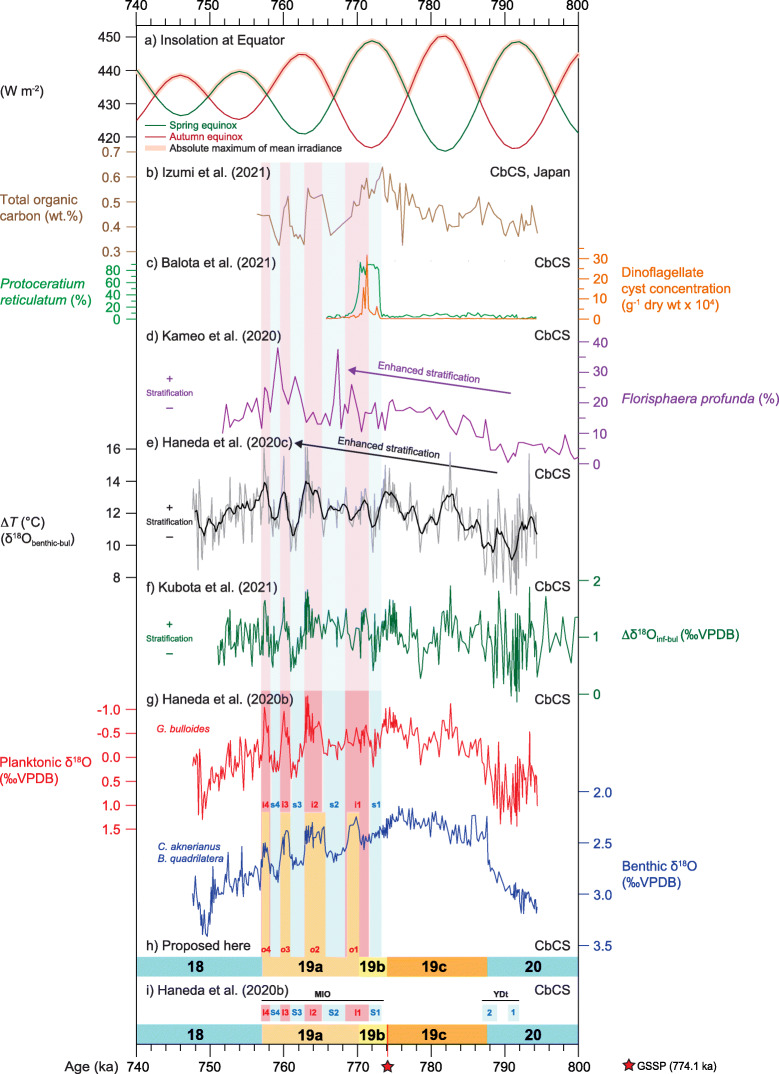
Chiba composite section (CbCS), Japan; records of Marine Isotope Stage 19 based on the age model of Suganuma et al. (2018). (**a**) Insolation at Equator in spring and autumn, and absolute maximum of mean irradiance (Laskar et al. 2004; Ferretti et al. 2015; Haneda et al. 2020b). (**b**) Total organic carbon wt.% (Izumi et al. 2021). (**c**) Percentage abundance of *Protoceratium reticulatum* cysts and total dinoflagellate cyst concentrations interpreted as a southward shift of the Kuroshio Extension during stadial s1 and early interstadial i1 (Balota et al. 2021). (**d**) Percentage abundance of calcareous nannofossil *Florisphaera profunda*, an indicator of near-surface ocean stratification (Kameo et al. 2020). (**e**) Profile of Δ*T* (δ^18^O_benthic_ minus δ^18^O_Globigerina bulloides_) as a measure of the vertical temperature gradient between surface and bottom waters (thin grey line) and 1000-year moving average (thicker black line) (Haneda et al. 2020b, 2020c). (**f**) Profile of Δδ^18^O_inf-bul_ (δ^18^O_Globorotalia inflata_ minus δ^18^O_Globigerina bulloides_) as an indicator of near-surface ocean stratification (Kubota et al. 2021). (**g**) Planktonic and benthic foraminiferal δ^18^O records (Haneda et al. 2020b). (**h**) The MIS 19 subdivisional scheme used here (Fig. 7): benthic marine isotope oscillations *o*1–*o*4 and interstadials i1–i4 are labelled in red, stadials s1–s4 are labelled in blue. (**i**) Substage classification and interstadial and stadial classification and labelling of Haneda et al. (2020b) where MIO = Millennial Isotopic Oscillation, showing MIO-Stadial 1 to 4 (MIO-S1 to MIO-S4), and MIO-Interstadial 1 to 4 (MIO-I1 to MIO-I4); YDt = Younger Dryas-type cooling subevent

The highly resolved δ^18^O record from the CbCS, Japan (Haneda et al. 2020b; Suganuma et al. in press; Fig. 4), has been subdivided into lettered substages such that four peaks identified in the upper part of MIS 19 are all assigned to MIS 19a (Fig. 11). This scheme follows the classification of Nomade et al. (2019).

It is evident from the foregoing that most records of MIS 19 naturally allow subdivision into two parts, an earlier relatively stable phase representing MIS 19c and occurring within one precession cycle, and a later phase (the inconsistently applied MIS 19b and MIS 19a) usually featuring three or four millennial-scale isotopically light peaks and occurring within a second precession cycle. There might be merit in dividing MIS 19 into just two substages separated by the current MIS 19c/b boundary as this most reasonably represents the inception of glaciation (Tzedakis et al. 2012a), and indeed Nomade et al. (2019) considered MIS 19b as the first bipolar seesaw oscillation. However, the tripartite subdivision first introduced by Bassinot et al. (1994) has become entrenched. The approach used here is therefore to follow the substage classification of Nomade et al. (2019) and Haneda et al. (2020b) in which MIS 19b is restricted to the first interval of high benthic isotopic values following MIS 19c.

#### Fine-scale subdivision of MIS 19

Millennial to centennial changes occurring within MIS 19 include both local and global signals. Recognizing events, for example of warming or drying, and numbering them consecutively without reference to their substage is the simplest approach. Sánchez-Goñi et al. (2016) applied constrained cluster analysis to subdivide their pollen record of Site U1385 into 20 numbered pollen biozones through MIS 19, and then used the relative abundances of Mediterranean pollen taxa to indicate intervals of Mediterranean forest contraction (Fig. 9c). Regattieri et al. (2019) in their study of the lacustrine Sulmona basin in Italy recognized nine events of reduced precipitation inferred from multiple paleoenvironmental proxies. These events were labelled I–IX and occur throughout MIS 19 (Fig. 10b).

An additional approach is the recognition of numbered stadials and interstadials. These have been used traditionally to describe cooler and warmer episodes within glacial cycles and are therefore climatic subdivisions. Their use in the Pleistocene (Penck and Brückner 1909) considerably predates that of marine isotope stages and substages. The two systems while obviously complementary are often based on different criteria. Stadials and interstadials are then more logically used alongside marine isotope substages than to subdivide them.

The benthic marine isotope record at Montalbano Jonico, Italy (Fig. 10d) shows at least three sharply defined lighter isotopic phases within MIS 19a, and Nomade et al. (2019) defined these as interstadial 19a-1, 19a-2, and 19a-3 (Fig. 10d). These interstadials are numbered in stratigraphically ascending rather than descending order, allowing them to be labelled consistently since a “fourth” interstadial at the end of MIS 19a is less pronounced and may not always be recognized. The Montalbano Jonico succession was deposited on the shelf in relatively shallow (~ 100–200 m) waters, and the benthic isotope record closely resembles other climatic proxies (Marino et al. 2020). Hence, in this case, the benthic isotopic record incorporates a localized climatic signal and serves to indicate stadial and interstadial conditions. Nomade et al. (2019) did not number adjacent stadials.

Haneda et al. (2020b) extended the scheme of Nomade et al. (2019) by subdividing the latter half of MIS 19 at the CbCS in Japan into both stadials and interstadials, labelling them as MIO-Stadial 1 to 4 (MIO-S1 to MIO-S4) and MIO-Interstadial 1 to 4 (MIO-I1 to MIO-I4), where MIO stands for Millennial Isotopic Oscillation (Fig. 11i). The first stadial was understandably assigned to the cooling event of MIS 19b that marks the inception of glaciation (Tzedakis et al. 2012a). These stadial–interstadial designations are based on the planktonic isotopic record which is largely a sea-surface temperature signal. It is essentially a climatic subdivision for which a stadial–interstadial designation is indeed appropriate. However, the MIS stage and substage boundaries at Chiba are based on the benthic foraminiferal δ^18^O record following the approach of Lisiecki and Raymo (2005) and Railsback et al. (2015), and consequently may not align precisely with the stadial–interstadial boundaries which are based on surface to near-surface (planktonic) properties. Hence, the first stadial (MIO-Stadial 1) begins just after the start of MIS 19b, and the first interstadial straddles the MIS 19b–a boundary (Fig. 11i).

The labelling scheme proposed here (Section 3.2.1; Fig. 7) extends and modifies the schemes of Nomade et al. (2019) and Haneda et al. (2020b). It treats separately the benthic isotopic record in which as many as four millennial-scale oscillations (MIS 19a-*o*1 to MIS 19a-*o*4) may be discerned, and the planktonic / terrestrial record in which as many as four stadials (MIS 19-s1 to MIS 19-s4) and four interstadials (MIS 19-i1 to MIS 19-i4) may be identified. It resolves the incompatibility between the benthic isotopic record which may contain a strong regional to global signal especially at deep-ocean sites and upon which MIS substages are often based, and the planktonic and terrestrial signals that emphasize more localized climatic variations and permit the most reliable characterization of stadials and interstadials. This approach can be used alongside informal climatic schemes that in some cases already facilitate the recognition of stadials and interstadials in the latter part of MIS 19 (e.g. Sánchez-Goñi et al. 2016; Regattieri et al. 2019; Figs. 9c and 10b).

Climatic fluctuations associated with deglaciation across Termination IX have also been labelled as stadials and interstadials using terminology developed for the last deglaciation (Mangerud et al. 1974; Björck et al. 1998). Giaccio et al. (2015) labelled an abrupt cold and dry interval from the Sulmona basin as a Younger Dryas-like event (Fig. 10b). Maiorano et al. (2016) applied the terms Heinrich-like, Bølling–Allerød-like, and Younger Dryas-like to similar climate oscillations recorded at the Montalbano Jonico section, and this terminology (Med-H_TIX_, Med-BA_TIX_, Med-YD_TIX_, referencing Termination IX in the Mediterranean) was continued by Marino et al. (2020; Fig. 10d). With respect to the CbCS in Japan, Suganuma et al. (2018) documented a single cooling phase they labelled as a Younger Dryas-type cooling event, and Haneda et al. (2020b) distinguished two closely separated cooling episodes they labelled as Younger Dryas-type cooling sub-events 1 and 2, abbreviated to YDt-1 and YDt-2 (Fig. 11i). These are effectively stadial–interstadial alternations but their precise expression is perhaps too uncertain at present to warrant a standardized terminology.

### Age model calibration of MIS 19 records

Lisiecki (undated) gives the bounding ages for MIS 19 as 790 and 761 ka, based on the Lisiecki and Raymo (2005) global benthic foraminiferal δ^18^O stack (LR04) which is tuned to the insolation curve for 65° N (Laskar et al. 1993). Several studies of MIS 19 have used the LR04 record as the tuning target for their age models, including Ferretti et al. (2015) for the central North Atlantic Site U1313 (Fig. 9b) and Sánchez-Goñi et al. (2016) for IODP Site U1385 off Portugal (Fig. 9c). However, a primary limitation of LR04 in this regard is its weak expression of millennial-scale oscillations that characterize MIS 19b–a (Fig. 13). It should also be noted that the LR04 stack while ostensibly a globally averaged record is in fact heavily biased towards the Atlantic and also contains a significant temperature component (Elderfield et al. 2012). Moreover, Pacific records lag the Atlantic by as much as ~ 4 kyr (Lisiecki and Raymo 2009; see Head 2019 for discussion). Suganuma et al. (2018) therefore used the sea-level proxy curve of ODP Site 1123 located on Chatham Ridge in the South Pacific (Elderfield et al. 2012; Fig. 4) as their tuning target for the CbCS in Japan, although this itself is tuned to the LR04 stack. An updated benthic foraminiferal δ^18^O data set (Haneda et al. 2020b; Suganuma et al. in press; Fig. 11g) prompted a slight readjustment of the stratigraphic positions of the stage and substage boundaries for the CbCS, as follows: 787.5 ka (MIS 20–19c), 773.9 ka (MIS 19c–19b), 770.1 ka (MIS 19b–19a), and 756.9 ka (MIS 19a–18) (Table 2). Nonetheless, the original astrochronological age model of Suganuma et al. (2018) was used in Haneda et al. (2020b) and Suganuma et al. (in press). This age model is subject to an uncertainty of about 5 kyr, allowing for an uncertainty of 4 kyr in the Lisiecki and Raymo (2005) target curve used for ODP Site 1123 (supplementary material in Elderfield et al. 2012), and an estimated 1 kyr uncertainty in tuning the Chiba record to the ODP Site 1123 sea-level curve.

**Table 2:** Extent and duration of MIS 19 and its substages and of full interglacial conditions. Ages and durations (in ka/kyr) are based on the age models published for each site

Author	Location	MIS19 duration	MIS19a ends	MIS19b ends	MIS19c ends	MIS19c duration	MS19c begins	Full Interglacial		
								ends	duration	begins
Lisiecki (undated)	LR04 global stack	29	761				790			
Tzedakis et al. (2012a)	ODP 983							~ 777.5	12.5–10.5	788
Ferretti et al. (2015)	IODP U1313							~ 779		
Sánchez-Goñi et al. (2016)	U1385	33	758	765	774	17	791	775	~12.5	787.5
Nomade et al. (2019)	Ideale section MBJ	30.5	757.0	772.7	773.9	13.7 ± 5	787.6	773.8	11.5 ± 3.4	785.3
Regattieri et al. (2019, fig. 9)	Sulmona	31	760	765	777	14	791	777	11	788
Haneda et al. (2020b)	Chiba	28.1	756.9	770.1	773.9	11.1	787.5	775.1	9.9	785.0
Toti et al. (2020)	ODP 976							~ 774	~ 8	~ 782

In addition to the limitations discussed above of using a single global stack such as LR04 as an alignment target, Lisiecki and Stern (2016) cautioned that the LR04 stack appears to be 1 to 2 kyr too young throughout the Pleistocene. The radiometric dating of interbedded tephras, correlation to radiometrically dated regional climatic events in speleothem records, and for the Mediterranean the use of sapropels and sapropel-like beds, should therefore be incorporated into age model construction wherever possible. Varve counting where available is also invaluable for precisely estimating the duration of events within MIS 19.

Nomade et al. (2019) implemented a hybrid chronology for the Ideal section of Montalbano Jonico in southern Italy that integrates both astronomical tie points, including a ghost sapropel tentatively representing insolation cycle 74 (Maiorano et al. 2016; Marino et al. 2020), and ^40^Ar/^39^Ar-dated tephra layers (Fig. 10d). Rigattieri et al. (2019) for the lacustrine Sulmona succession in central Italy used an age model based exclusively on ^40^Ar/^39^Ar-dated tephra layers, six of these occurring through an 805–753-ka interval spanning MIS 19 (Fig. 10b). The lacustrine deposits at Piànico-Sèllere in northern Italy allow a floating varve chronology to be combined with a K/Ar-dated tephra layer (Pinti et al. 2001; Roulleau et al. 2009; Nomade et al. 2019; Fig. 10c). All these approaches are subject to uncertainties, some of which cannot presently be estimated.

Table 2 shows the age and duration of MIS 19c, 19b, and 19a for each of the sites discussed using their own time scales to illustrate the variation recorded, which reflects tuning uncertainties as well as local and regional influences superimposed on a global ice volume signal. One example serves to illustrate these uncertainties. Interstadial i1 (MIS 19a-1 in the scheme of Nomade et al. 2019) is sharply defined in Italy with its bounding transitions concluded within 200 years (Fig. 10b–d). Comparing its duration based on the published time scale of each site, it is ~ 4.3 kyr at Sulmona (Regattieri et al. 2019, fig. 5), 3.2 kyr at Montalbano Jonico (Nomade et al. 2019), and 4.9 kyr at Piànico-Sèllere (Nomade et al. 2019). As noted by Nomade et al. (2019), these discrepancies illustrate some of the challenges in refining the age model for the latter part of MIS 19.

**Fig. 12:**
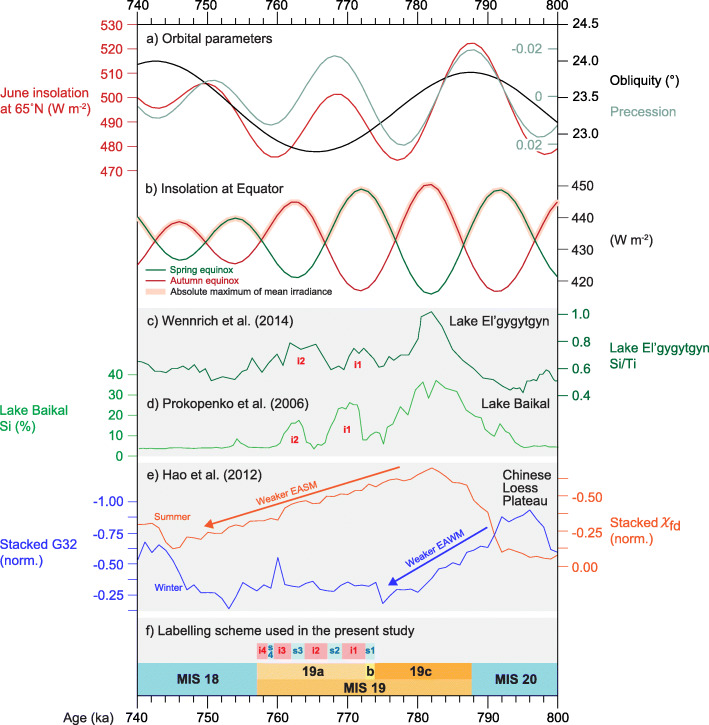
Selected Asian records of Marine Isotope Stage 19. (**a**) Insolation at 65° N in June, and precession and obliquity parameters. (**b**) Insolation at Equator in spring and autumn, and absolute maximum of mean irradiance (Laskar et al. 2004; Ferretti et al. 2015; Haneda et al. 2020b). (**c**) Lake El’gygytgyn, northeast Siberia: XRF core scanning-derived Si/Ti ratio (Wennrich et al. 2014). (**d**) Lake Baikal, southern Siberia: Biogenic silica contents (Prokopenko et al. 2006). (**e**) Normalized Yimaguan and Luochuan (China) stacked loess–palaeosol proxy records for East Asian Summer Monsoon (EASM; frequency-dependent magnetic susceptibility, orange line) and East Asian Winter Monsoon (EAWM; > 32 μm particle content, blue line) (suppl. fig. 12 of Hao et al., 2012). (**f**) The MIS 19 subdivisional scheme used here (Fig. 7): interstadials i1 and i2 are labelled in red. All records are plotted on their own published time scales

Other sites have been studied at lower stratigraphic resolution through MIS 19. Lake Baikal in southeastern Siberia (~ 53°N, Figs. 4 and 12d) represents an area with the highest sensitivity to insolation forcing on Earth, owing to its central position within Asia. By correlating biogenic silica peaks, representing lake productivity maxima, with precessional cycles (Laskar et al. 2004), an astronomically tuned composite record of the biogenic silica was obtained over the entire Pleistocene. Magnetostratigraphic boundaries enabled the cross-checking of this chronology (Prokopenko et al. 2006). The Holocene–Pliocene record of Lake El’gygytgyn (67° 30′ N, 172° 05′ E; Figs. 4 and 12c), located in the Far East Russian Arctic, was dated using a combination of magnetostratigraphic reversals and palaeoclimatic records tuned to summer insolation at 65° N (Laskar et al. 2004) and to the Lisiecki and Raymo (2005) LR04 global stack (Nowaczyk et al. 2013). It is worth reiterating here concerns about using the LR04 global stack for this kind of tuning (Lisiecki and Stern, 2016).

### The climatic evolution of MIS 19

**Fig. 13:**
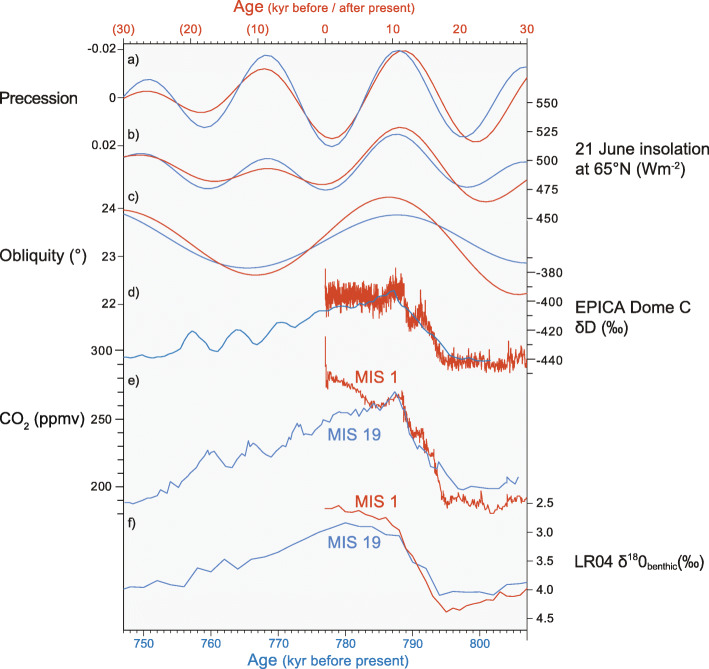
Orbital and climatic characteristics of MIS 19 (blue) and the present interglacial MIS 1 (red) compared. (**a**–**c**) Orbital configurations and (**b**) insolation for MIS 19 and past and future MIS 1 (Laskar et al. 2004). (**d**) δD composition of ice in the EPICA Dome C ice core as a proxy for Antarctic surface temperature (Jouzel et al. 2007). (**e**) Antarctic ice-core CO_2_ values from the 0–800 kyr BP composite record of Bereiter et al. (2015, supplementary data) and show MIS 1 and MIS 19 in close alignment through the Early Holocene. (**f**) LR04 benthic stack of Lisiecki and Raymo (2005) showing lighter values for MIS 1, beginning ~ 11,000 years before present. Adapted from fig. 3 of Tzedakis et al. (2012b)

MIS 19 has been studied intensively owing to the close similarity between its substage c and the present interglacial with respect to orbital configuration, rapid deglaciation history, and early peak Antarctic temperatures (Tzedakis et al. 2012b; Fig. 13). This similarity allows unambiguous alignment of MIS 19c with the present interglacial, thereby offering insights into our future climate (Tzedakis et al. 2012b).

Earlier overviews of the climatic evolution of MIS 19 are given by Tzedakis et al. (2012a, b) and most recently with inter-site comparisons by Suganuma et al. (2018, in press), Nomade et al. (2019), Regattieri et al. (2019), and Haneda et al. (2020b). Sites yielding highly resolved paleoclimatic records are listed in Table 3 and show a concentration of sites in the northern hemisphere. The modelling study of Vavrus et al. (2018) adds spatial detail to this picture.

**Table 3:** Sites from which highly resolved paleoclimatic records of MIS 19 are available

Site	Location	Latitude, longitude	Deposition	Selected publications
ODP Site 983	Gardar Drift, North Atlantic	60.4° N, 23.6° W	Marine	Kleiven et al. (2011), Tzedakis et al. (2012b)
IODP Site U1313	Central North Atlantic	41° 00′ N, 32° 58′ W	Marine	Ferretti et al. (2015), Emanuele et al. (2015)
IODP Site U1385	Eastern North Atlantic off SW Portugal	37° 34.285′ N, 10° 7.562′ W	Marine	Sánchez-Goñi et al. (2016)
ODP Site 976	Alboran Sea, western Mediterranean	36° 12.3′ N, 4° 18.8′ W	Marine	Toti et al. (2020)
Valle di Manche	Calabria, southern Italy	39° 05′ 39″ N, 16° 55′ 13″ E	Marine	Capraro et al. (2004, 2005, 2019), Azzarone et al. (2018), Macrì et al. (2018), Rossi et al. (2018)
Montalbano Jonico	Basilicata, southern Italy	40° 17′ 30″ N, 16° 33′ 11″ E	Marine	Bertini et al. (2015), Marino et al. (2015, 2020), Maiorano et al. (2016), Simon et al. (2017), Nomade et al. (2019)’
Sulmona basin	Central Italy	42° 09′ 07″ N, 13° 49′ 15″ E	Lacustrine	Giaccio et al. (2013, 2015), Regattieri et al. (2019)
Piànico-Sèllere basin^a^	Northern Italy	45° 48′ N, 10° 02′ E	Lacustrine	Moscariello et al. (2000), Scardia and Muttoni (2009), Roulleau et al. (2009)
Chiba composite section	East-central Japan	35° 16′ 51″ N, 140° 07′ 28″ E to 35° 18′ 38″ N, 140° 11′ 50″ E	Marine	Suganuma et al. (2015, 2018, in press), Haneda et al. (2020b), Kameo et al. (2020), Balota et al. (2021), Izumi et al. (2021), Kubota et al. (2021)
EPICA Dome C	Antarctica	75° 06′ S, 123° 21′ E	Ice sheet	Jouzel et al. (2007), Bazin et al. (2013), Bereiter et al. (2015)

^a^The Piànico-Sèllere site was assigned to MIS 19 only in 2001 (Pinti et al. 2001, 2007), having previously been considered much younger

MIS 19 compares with both MIS 11 and the present interglacial in having a reduced-amplitude 400 ky eccentricity cycle and consequent suppression of precessional forcing (Fig. 2). Precession is in phase for all three interglacials. However, whereas the obliquity peak closely aligns with the precession minimum for both MIS 19c and the present interglacial, it leads the precession minimum by about 9 kyr in MIS 11. As a result, June insolation at 65° N increases more slowly for MIS 11 than for MIS 19c and the present interglacial (fig. 6 of Tzedakis 2010). MIS 19c is therefore the closest orbital analogue for the present interglacial and even though the amplitude of obliquity is lower for MIS 19c the alignment of their onset is unambiguous (Tzedakis et al. 2012b). This close similarity will begin to diverge in the future, as the amplitude of precession will decline more strongly than for late MIS 19, and June insolation at 65° N will be lower (Fig. 13).

Although the phasing between precession and obliquity are closely similar for MIS 19c and the present interglacial, with the obliquity maximum close to the precession minimum, obliquity during MIS 19c increases less rapidly and hence to a lower amplitude than during the beginning of the present interglacial. Moreover, the LR04 foraminiferal isotopic record shows lighter peak values for MIS 1 (figs. 6 and 7 of Tzedakis 2010; Fig. 13) and agrees with observations from the CbCS that temperatures were cooler during MIS 19 than today (Suganuma et al. 2018). Ganopolski et al. (2016) proposed that higher CO_2_ levels of around 280 ppm during the pre-industrial Late Holocene explain this temperature difference, and Studer et al. (2018) discussed reasons for the exceptional rise in CO_2_ from 8 ka (Middle Holocene) onwards (Fig. 13). However, for the Early Holocene, CO_2_ levels reached a maximum of only 270 ppm which is very close to the 269 ppm maximum for MIS 19 based on corrected CO_2_ records for the EPICA Dome C core (Bereiter et al. 2015). Indeed, Early Holocene CO_2_ levels track those of MIS 19 closely and do not provide an explanation for a pronounced trend to lighter values seen in the δ^18^O stack of Lisieck and Raymo (2005) (Fig. 13). MIS 19c therefore presents a good analogue at least for the Early Holocene based on CO_2_ as well as orbital criteria. It should be noted that residual global ice volume might have been higher during MIS 19 than MIS 1 (Elderfield et al. 2012; Regattieri et al. 2019; Vavrus et al. 2018). This may have increased climate sensitivity during MIS 19 given the nonlinear relationship between astronomical forcing and ice volume during the Quaternary (Past Interglacials Working Group of PAGES 2016) and the fact that polar ice volume provides one of the most important feedback mechanisms in the climate response to radiative forcing (Berger et al. 2017; Westerhold et al. 2020).

#### MIS 20–19 transition

There is widespread evidence of climatic and oceanographic instability during late MIS 20 and across Termination IX. A Younger Dryas-type cooling event interrupts the deglaciation of Termination IX in several records, notably at Montalbano Jonico (Maiorano et al. 2016; Simon et al. 2017; Marino et al. 2020) and Sulmona (Giaccio et al. 2015; Regattieri et al. 2019) in Italy, and the CbCS in Japan (Suganuma et al. 2018; Haneda et al. 2020b), and is dated at around 785–790 ka. A similar cooling event is also recorded in the Lake Baikal succession in Russia (Prokopenko et al. 2006; Fig. 12d) and elsewhere. At the CbCS, the dinoflagellate cyst record registers a rapid retreat of the Subpolar Front at 789.3 ka, indicating warming around 2000 years before the end of MIS 20 (Balota et al. 2021). At IODP Site U1313 in the central North Atlantic, the calcareous nannofossil record indicates a return of the North Atlantic Current to this site at 793 ka in association with Termination IX (Emanuele et al. 2015).

At Montalbano Jonico, a cool–warm–cool succession begins with a stadial, labelled a Heinrich-like episode, near the end of MIS 20 (Med-H_TIX_ of Marino et al. 2020; Fig. 10d) that may be coherent with a freshwater pulse at IODP Site U1385 off SW Portugal (Sánchez-Goñi et al. 2016; Fig. 9c). This suggests a direct Mediterranean response to climate dynamics in the high-latitude North Atlantic (Marino et al. 2020). Cooling at Montalbano Jonico is followed by a Bølling-Allerød-like warming phase (Med-BA_TIX_) and then Younger Dryas-like cooling (Med-YD_TIX_) prior to final rapid warming early in MIS 19c. This same succession is observed at Sulmona (Regattieri et al. 2019; Fig. 10b) and in Core KC01B in the central Mediterranean Sea (Trotta et al. 2019; Marino et al. 2020; Fig. 4), suggesting a pattern of oscillations that is at least regional in extent.

A subsequent brief climatic reversal at ~ 785 ka observed in the pollen record of ODP Site 976 in the western Mediterranean (Toti et al. 2020; Fig. 10e) and a cooling event documented at IODP Site U1385 off Portugal (Sánchez-Goñi et al. 2016; Fig. 9c) both occur just before the plateau of lightest isotope values for MIS 19c and may reflect the same event.

A combination of obliquity phasing with low precessional forcing amplitude may have been a precondition for the instability seen across the MIS 20–19 transition (Suganuma et al. 2018). The actual trigger likely reflects a short-term disruption of the Atlantic meridional overturning circulation (AMOC) and would have connected to the Pacific through shifts in the Intertropical Convergence Zone (ITCZ) (Haneda et al. 2020b; Fig. 4). The presence of two closely separated Younger Dryas-type subevents at the CbCS (Haneda et al. 2020b; Fig. 11i) attests to the complexity of processes in operation at the global scale.

#### MIS 19c

MIS 19c has among the lightest isotopic values and spans full interglacial conditions, the duration of which holds considerable interest in assessing the natural length of our own interglacial. MIS 19c extends from around 791–787.5 ka to the expansion of ice sheets (glacial inception) at 774–777 ka and represents a more stable episode mostly coinciding with full interglacial conditions (Table 2).

In spite of weak eccentricity at this time, planktonic and benthic foraminiferal isotope data for central North Atlantic Site U1313 (Fig. 9b) reflect variation concentrated in the half-precession bandwidth (~ 11 kyr), and also quarter-precession bandwidth for the benthic foraminiferal isotope data, indicating low-latitude insolation forcing particularly when the amplitude of precession is at its greatest, which is during MIS 19c (Ferretti et al. 2015). The second harmonic of precession occurs when the perihelion of Earth’s orbit coincides with the spring or autumn equinox (fig. 10 of Ferretti et al. 2015; Fig. 9a). Dinoflagellate cyst analysis indicates peak interglacial conditions between 790.5 and 784.0 during which time Site U1313 was fully under the influence of the subtropical gyre (Abomriga 2018). Coccolithophore assemblages reflect modern-type warmer North Atlantic Transitional Waters between ~ 788 and 782 ka, with glacial inception occurring at ~ 779 ka (Ferretti et al. 2015; Emanuele et al. 2015). Glacial inception appears to have occurred quite early but with enhanced iceberg discharges from ~ 776 ka onwards suggesting multiple ice-sheet calving events (Ferretti et al. 2015).

IODP Site U1385 off southwestern Portugal (Sánchez-Goñi et al. 2016; Fig. 9c) has a detailed pollen record of vegetation development across MIS 19. Full interglacial conditions (the Tajo interglacial) as determined by this pollen record extend from 787.5 to 775 ka. MIS 19c extends to stadial s1 (Fig. 9c) as identified by correlating to the Sulmona basin record (Regattieri et al., 2019; fig. 9i in Nomade et al. 2019; Fig. 10b). Significantly, Mediterranean forest growth during MIS 19c was interrupted by three pronounced contractions (fig. 5 of Sánchez-Goñi et al. 2016; Fig. 9c) that represent cooling and drying events not significantly reflected in the proxies for sea-surface freshwater input or temperature, which are relatively stable and warm through MIS 19c. This decoupling of terrestrial and marine climate through MIS 19c implies that the westerlies supplying moisture to southern Portugal through most of MIS 19c were periodically diverted northwards along with moisture that would have contributed to the growth of high-latitude ice sheets. These were expanding progressively through MIS 19c, aided by decreasing boreal summer insolation. These forest contraction events occur throughout MIS 19 with a 5 kyr periodicity and appear to represent a response to the fourth harmonic of precession, implying as with central North Atlantic Site U1313 the influence of low-latitude insolation forcing at this location. These decoupling events are not restricted to MIS 19 as they occur at other times during the Quaternary along the European margin (Sánchez Goñi et al., 2018).

Within the Mediterranean region, ODP Site 976 in the Alboran Sea (Fig. 4) is influenced directly by Atlantic inflow and has yielded detailed pollen, coccolithophore, foraminiferal assemblage, and planktonic foraminiferal δ^18^O records through MIS 19 (Toti et al. 2020; Fig. 10e). Millennial-scale climate oscillations occur throughout MIS 19c and are registered synchronously in both marine and terrestrial proxies. Brief episodes of forest contraction centred at 781, 780, 777, and 775 ka through MIS 19c attest to short-term climate fluctuations (less humid winter conditions) presumably caused by the periodic northward deflection of westerlies as proposed by Sánchez-Goñi et al. (2016) for Site U1385 off Portugal. Similar fluctuations are observed in the Sulmona succession in Italy, suggesting that the entire western Mediterranean was affected by precession-driven episodes of drought through MIS 19c (Toti et al. 2020, and see below).

In Italy, the marine Montalbano Jonico composite section and the lacustrine Sulmona basin succession have provided exceptionally detailed and well constrained records through MIS 19. Montalbano Jonico includes foraminiferal isotopes, various marine proxies, and pollen (Bertini et al. 2015; Nomade et al. 2019; Marino et al. 2020; Fig. 10d). The onset of full interglacial conditions shortly after the start of MIS 19c is marked by a faint sapropel-like feature (“ghost sapropel”) assigned tentatively to i-cycle 74 by Maiorano et al. (2016) which has a midpoint age of 784 ka based on a phase lag of 3 kyr relative to maximum insolation in June at 65° N (Lourens 2004). Emeis et al. (2000) assigned a comparable red horizon from the eastern Mediterranean to i-cycle 74 although this might represent an obliquity maximum rather than a precession minimum (Konijnendijk et al. 2014), although the two are in phase for MIS 19c. This recalls the often overlooked influence of high-latitude obliquity on Mediterranean climate (Konijnendijk et al. 2014) even in the absence of high-latitude ice sheet dynamics (Bosmans et al. 2015). The ghost sapropel at Montalbano Jonico lasted for about 2.5 kyr, and represents an interval of water-column stratification resulting from freshwater inflow related to strengthened North African summer monsoon conditions during the insolation maximum (Nomade et al. 2019; Marino et al. 2020). The presence at ODP Site 975 in the western Mediterranean (Fig. 4) of an organic-rich layer within MIS 19c suggests this to be a basin-wide phenomenon (Quivelli et al. 2020).

Pollen records from the Ideale section at Montalbano Jonico evidence an interval of full interglacial conditions (climatic optimum) marked by the expansion of temperate forests. These forests were dominated by broad-leaved trees and indicate a warm and relatively humid climate. This climatic optimum extends from the sapropel-like layer to the top of MIS 19c at ~ 774 ka, with a duration of 11.5 ± 3.4 kyr (Nomade et al. 2019; Fig. 10d). Three mesothermic forest expansions are recognized and are almost concurrent with higher sea-surface temperature phases as reflected by alkenone SST reconstructions and the abundance of calcareous nannofossils. These phases are labelled I, II, and III in Marino et al. (2020; Fig. 10d). Spectral analysis also shows climate oscillations occurring with a periodicity of about 5.4 kyr throughout MIS 19 although these are dampened in MIS 19c (Nomade et al. 2019). The increasing benthic foraminiferal isotope values towards the end of MIS 19c suggest a strong global component in spite of the shallow (not more than 180–200 m deep) marine setting. The benthic foraminiferal record registers a maximum depth between ~ 778.1 and 773.4 ka which spans the 774-ka timing of maximum global sea level given by Elderfield et al. (2012).

The lacustrine Sulmona basin record (Regattieri et al. 2019; Fig. 10b) is just 295 km to the northwest of Montalbano Jonico (Nomade et al. 2019) and is based on a ^40^Ar/^39^Ar timescale fully independent of orbital tuning and with a mean uncertainty of ± 2.6 kyr. A duration of 11 kyr for full interglacial conditions agrees with the 11.5 ± 3.4 kyr duration recorded for the Ideale section at Montalbano Jonico. The stable isotope record at Sulmona has a temporal resolution of ~ 60 years, allowing MIS 19c to be analysed in exceptional detail. Rapidly increasing precipitation after 788 ka, reflecting deglaciation, reached a peak at ~ 786 ka but was interrupted by a prominent 0.8 kyr-long drier interval starting at 785 ka (event I of Regattieri et al. 2019) and speculated to be analagous with the 8.2 ka event in the Holocene. This is followed by additional events of increasing dryness. Regattieri et al. (2019) linked event I with a reduction in deep-water ventilation at ODP Site 983 (Fig. 8c), implying a connection with a brief interruption of the AMOC. These authors therefore proposed that these drying events in MIS 19c were causally linked to deep hydrography in the northern North Atlantic although were not able to tie them specifically to precession forcing.

A detailed pollen record from the CbCS in Japan shows a well-defined rise and fall in deciduous broad-leaved trees between 785.0 and 775.1 ka (pollen subzone CbCS-2a in fig. 7 of Suganuma et al. 2018), suggesting 9.9 kyr for the duration of full interglacial conditions at this site. The benthic foraminiferal δ^18^O record (Fig. 11g) shows a steep rise to lighter values at the beginning of MIS 19c and a more gradual decline towards the end, with otherwise relatively little variation (Haneda et al. 2020b; Suganuma et al. in press). In contrast, *G. bulloides* (planktonic) foraminiferal δ^18^O values show considerable fluctuations (Fig. 11g), and a high-resolution study of the dinoflagellate cysts across MIS 19c reveals instability and latitudinal shifts in the Kuroshio Extension system at this site (Balota et al. 2021). This instability presumably reflects the close proximity of the CbCS to the convergence of the warm Kuroshio and cold Oyashio currents that forms an extreme hydrographic gradient in this part of the western North Pacific (Kubota et al. 2021). A spectral and wavelet analysis of the planktonic foraminiferal δ^18^O record and an index for water-column stratification reveal 9.6 kyr cycles throughout MIS 19 but expressed particularly strongly through MIS 19c (Haneda et al. 2020b). This is interpreted as the second harmonic of precession. As with North Atlantic Site U1313 (Ferretti et al. 2015), it seems related to equatorial insolation forcing and similarly appears to have been greatest during MIS 19c when precession at the Equator was at its highest amplitude (Fig. 11a).

#### MIS 19b and a

A critical feature of MIS 19b and 19a, together representing approximately the second half of MIS 19, is the establishment of three or four stadial/interstadial alternations broadly coinciding with a second precessional minimum that is in antiphase with obliquity, resulting in a damped insolation peak (Fig. 7a). This is transposed onto a longer-term trend of increasing global ice volume. Several mechanisms have been proposed for the bistability in MIS 19b–a.

Tzedakis et al. (2012b) posited that the termination of full interglacial conditions at the end of MIS 19c would have coincided with the expansion of ice sheets leading to iceberg discharges into the North Atlantic and hence disruption of the AMOC. This in turn will have led, after some delay, to warming over Antarctica by means of the thermal bipolar seesaw mechanism (Stocker and Johnsen 2003). This happens when heat normally transported northward into the North Atlantic Ocean instead accumulates to the south in the global interior ocean, resulting after a short delay in its advection southwards into the Southern Ocean (Pedro et al. 2018). From here it then causes warming over Antarctica. Our understanding of the thermal bipolar seesaw derives from the study of Dansgaard–Oerschger events during the last glaciation (and their Holocene equivalents called Bond cycles) in Greenland ice cores and their antiphase relationship with their equivalent Antarctic Isotope Maxima (AIMs) in Antarctic ice cores. While this teleconnection is well documented, the mechanism controlling the frequency of Dansgaard–Oeschger events is not known. Tzedakis et al. (2012b) examined published records of MIS 19 from ODP Site 983 on the Gardar Drift, northern North Atlantic (Channell and Kleiven 2000; Kleiven et al. 2011; Fig. 8c). They noted that for the MIS 19b–a interval, three minima in the planktonic foraminiferal δ^18^O records match temperature peaks in the Antarctic ice-core record and that minima in the planktonic and benthic foraminiferal δ^18^O records are phase-shifted in a manner that invokes the thermal seesaw. Moreover, peaks of ice-rafted debris at ODP Site 983 occurring after the end of MIS 19c (Fig. 8c) seem to support the contention that ice-sheet calving had triggered AMOC disruption, leading to the proposal that thermal seesaw bistability caused the pronounced minima in the second half of MIS 19 at ODP Site 983. Further support for this mechanism comes from the sortable silt and benthic δ^13^C records at Site 983 (Fig. 8c) that point to a slowdown of North Atlantic Deepwater Formation which is an essential component of the AMOC (Kleiven et al. 2011). A characteristic feature of the planktonic foraminiferal δ^18^O record is the rectangular-shaped waveform that seems to result from abrupt ice-sheet calving followed by the rapid return to strength of the AMOC. This contrasts with the much slower response of Antarctica via AMOC perturbations and the bipolar seesaw which translates via deep-water advection to a time lagged and v-shaped corresponding benthic record (Shackleton et al. 2000; Tzedakis et al. 2012b).

Ferretti et al. (2015) in their analysis of foraminiferal isotopes (Fig. 9b) and alkenones from IODP U1313 in the central North Atlantic found strong variability at frequencies of ~ 11 kyr, in both surface and deep-water records, and 5.8 kyr in the benthic oxygen signal suggesting forcing mediated by the second and fourth harmonics of precession. Because the harmonics of precession are important features of insolation in the tropics, resulting in two insolation peaks for every precessional cycle (Fig. 9a), this implies that low-latitude astronomical forcing and other processes are important drivers of millennial-scale climate variability even at a time when the effects of precession on insolation are subdued (Ferretti et al. 2015).

At IODP Site U1385 off southwestern Portugal, a forest contraction beginning at 775 ka marks the end of the Tajo interglacial (Sánchez-Goñi et al. 2016; Fig. 9c). Glacial inception terminating MIS 19c is marked by a significant marine cooling event at 769 ka on the time scale of Sánchez-Goñi et al. (2016), based on correlation with the independently dated Sulmona basin record in Italy by Regatierri et al. (2019). This event, which also represents a forest contraction, is dated at ~ 772 ka on the Sulmona time scale. It represents MIS 19b and is labelled as stadial s1 on Fig. 9c. This stadial is followed by a further two. All three stadials represent forest contractions and reflect cooling and drying episodes as with MIS 19c but are accompanied by heavier planktonic and benthic foraminiferal δ^18^O values. Moreover, alkenone results indicate both cooling and freshening of the surface waters (Fig. 9c) and may align with three IRD peaks at ODP Site 983 on the Gardar Drift in the subpolar North Atlantic (Fig. 8c). With the expansion of ice sheets, a threshold must have been crossed allowing the triggering of successive iceberg discharges and accompanying freshening and cooling of the surface waters off Portugal (Sánchez-Goñi et al. 2016). Spectral analysis of records from Site U1385 reveals ~ 5 kyr periodicity throughout MIS 19, suggesting the fourth harmonic of precession and hence the influence of equatorial insolation. The relatively high ice-volume baseline conditions for MIS 19 may have increased the sensitivity of this interglacial to high-frequency climate oscillations. Sánchez-Goñi et al. (2016) contrasted the dominant ~ 5 kyr cyclicity in global records of MIS 19 with the dominant 2.5 kyr cyclicity extracted from the Mediterranean forest pollen record of MIS 1 (Sánchez-Goñi et al. 2016, fig. 8a), calling into question some of the accepted similarities between the two marine isotope stages.

At Montalbano Jonico, MIS 19c ends at ~ 774 ka and is succeeded by four abrupt oscillations (*o*1–*o*4) that define two stable states between higher and lower benthic foraminiferal δ^18^O values (Nomade et al. 2019; Fig. 10d). The transitions from one state to the other took less than 200 years. These oscillations almost precisely coincide with alkenone SST reconstructions and are supported by simultaneous expansions and contractions of the mesothermic forest (warm/wet versus cool/dry climates), allowing the recognition of three interstadials in Nomade et al. (2019) and Marino et al. (2020) and four here (i1–i4; Fig. 10d).

These interstadials and their intervening stadials are superimposed on a longer-term trend of dryer and cooler climates through the latter part of MIS 19. They arise and decline with the same distinctive rapidity observed in the high-resolution records at Sulmona and Piànico-Sèllere, also in Italy. They occur within a single precession cycle and are the amplified part of a 5–6 kyr cyclicity detectable throughout MIS 19. Nomade et al. (2019) emphasized that while a direct linkage of these oscillations to Northern Hemisphere ice sheet dynamics and North Atlantic IRD events is clear, local factors are also needed to explain the large amplitude of these oscillations and their abrupt nature. The influence of the African monsoon on Mediterranean climate is already well illustrated by the development of sapropels during precession minima (insolation maxima). Nomade et al. (2019) therefore raised the possibility that interstadials might reflect oscillatory northward shifts of the ITCZ (Fig. 4) over the Mediterranean region. This would have brought increased summer moisture and temperature during the African monsoon. Two lines of evidence support this connection. Firstly, the interstadials, along with MIS 19c, are represented by dark grey silty clays, whereas the intervening stadials and MIS 19b are light grey. The darker clays have more negative δ^13^C values and indicate reduced water-column ventilation which may be explained by freshwater input through monsoon rains in the same way that sapropels are formed. As noted by Nomade et al. (2019), the three most pronounced interstadials closely match the three methane peaks in the EPICA Dome C ice-core record. The West African monsoon has a major effect on global methane production (Kleinen et al. 2020) and provides a potential link between the Mediterranean interstadials and Antarctic ice-core methane during MIS 19a. Nomade et al. (2019) therefore suggested that the wet/warm oscillations found at Montalbano Jonico but also at Sulmona and Piànico-Sèllere correspond to worldwide climatic phenomena associated with the tropical monsoon regime modulated by latitudinal shifts in the ITCZ.

In the Sulmona basin record (Giaccio et al. 2013, 2015; Regattieri et al. 2019), at least three interstadials can be recognized within the second half of MIS 19, and these clearly correlate to interstadials i1, i2, and i3 at Montalbano Jonico, with a fourth possibly also recognizable at Sulmona (Fig. 10b). Regattieri et al. (2019) noted the concordance between their reduced-precipitation events VII, IIX [sic], and IX (stadials s1, s2, and s3) and the subpolar record of IRD and linked these events to disruptions of the AMOC. Hence, whereas Nomade et al. (2019) proposed northward shifts in the ITCZ and increased influence of the monsoon to explain millennial-scale shifts in climate at Montalbano Jonico, Regattieri et al. (2019) invoked the direct influence of AMOC weakening on the Sulmona record, noting numerous examples of high-latitude forcing on the Mediterranean climate during the Quaternary. The influence of the African monsoon on Mediterranean hydrography nonetheless remains uncontestable.

ODP Site 976 in the Alboran Sea (Toti et al. 2020; Fig. 4) is situated close to the Strait of Gibralter and is strongly influenced by hydrographic exchanges with the North Atlantic. Its pattern of stadial–interstadial alternations compares closely with that recorded in other Mediterranean sites (Fig. 10) and with Site U1385 off southwestern Portugal (Fig. 9c). Cyclic northern shifts of the ITCZ explain the increased winter precipitation needed for the expansion of Mediterranean forests during interstadials (Nomade et al. 2019; Toti et al. 2020). These northern shifts also facilitated the inflow of warm waters from the Azores Current into the Alboran Sea, as evidenced by increases in the abundances of warm water coccolithophore and foraminiferal taxa. Stadials conversely are characterized by increases in polar to subpolar foraminifera indicating the inflow of reduced-salinity subpolar waters from the North Atlantic. ODP Site 976 has therefore recorded the combined influences of North Atlantic inflow and atmospheric processes throughout MIS 19.

Sites located beyond the direct influence of the North Atlantic circulation and the AMOC are crucial in determining additional factors that might have driven the development of MIS 19, especially the stadial/interstadial oscillations during MIS 19b–a. The CbCS provides the most detailed MIS 19 record in the Pacific realm (Suganuma et al. 2018, in press; Haneda et al. 2020b; Kameo et al. 2020; Izumi et al. 2021, Balota et al. 2021; Kubota et al. 2021; Fig. 11). Here, climatic oscillations characterizing MIS 19b–a are well defined and reveal harmonics of precession that point to low-latitude forcing. Latitudinal shifts of the ITCZ (Haneda et al. 2020b) along with fluctuations in the Siberian High–Aleutian Low atmospheric system that controls the East Asian winter monsoon and Westerly Jet (Suganuma et al. 2018; Kubota et al. 2021) seem to have been driving factors. Oscillations of the ITCZ in particular would explain the similar stadial–interstadial pattern recorded at the CbCS and the North Atlantic–Mediterranean sites (Haneda et al. 2020b). The CbCS is discussed in detail in the next section.

Further evidence for climatic oscillations in the latter part of MIS 19 is found in the higher-latitude biogenic silica records of Lake Baikal (Prokopenko et al. 2006) and Si/Ti records of Lake El’gygytgyn (Wennrich et al. 2014). These records reflect elevated diatom production during the spring–fall and show pronounced interstadials i1 and i2 (Fig. 12c, d), recalling similar oscillations from the CbCS, North Atlantic Ocean, Mediterranean region, and Antarctic ice-core records. The oscillations at Lake Baikal are paced by the harmonics of precession, the influence of precession reaching Lake Baikal at 51°–53° N (although declining with increasing latitude; Prokopenko et al. 2006). The pronounced oscillations in MIS 19b–a may represent amplifications connected to global ice volume that had been increasing since the latter part of MIS 19c.

The lake El’gygytgyn record of northeastern Siberia (Wennrich et al. 2014; Fig. 12c), while showing climate oscillations similar to those at Chiba during MIS 19b–a, should have been influenced directly by the northern Siberia ice sheet at this time (Vavrus et al. 2018). As with the IRD record of ODP Site 983 just south of Iceland (Fig. 8c), the mechanisms driving these high-latitude oscillations are not well understood but appear phase linked across the northern hemisphere and suggest high-latitude atmospheric teleconnections, as discussed in Section 4.2 below.

## The Chiba composite section and GSSP

**Fig. 14:**
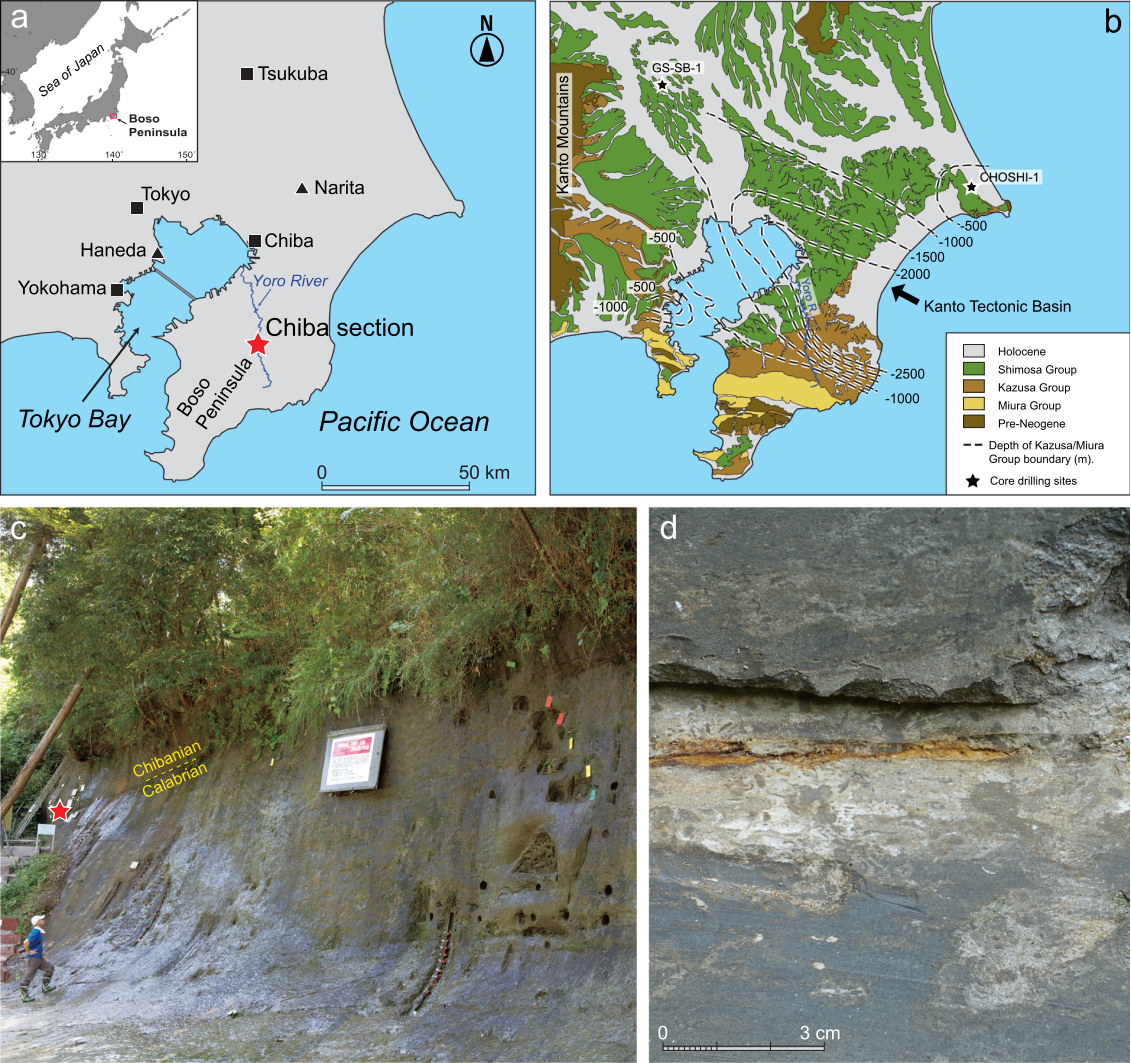
The Chiba section, Japan. (**a**) Location of the Chiba section on the Boso Peninsula showing major cities. (**b**) Schematic geology of the Kanto area showing the depth (metres below sea level) of the base of the Kazusa Group (from fig. 1 of Kazaoka et al. 2015). (**c**) Chiba section showing faint but discernible parallel bedding in this massive siltstone unit; the location of the GSSP is indicated by a red star (from fig. 10 of Suganuma et al. in press). (**d**) Detail of the Ontake-Byakubi-E (Byk-E) tephra bed in the Chiba section at the position of the GSSP, showing bioturbation. The GSSP is located at the base of the tephra bed (photo by the author). The GSSP has an astronomical age of 774.1 ± 5 ka and is 1.1 m below the directional midpoint of the Matuyama–Brunhes paleomagnetic reversal (Suganuma et al. in press)

The Chiba section, located in the central part of the Boso Peninsula and within the Chiba Prefecture, contains the GSSP (35° 17′ 39.6″ N, 140° 08′ 47.6″ E) for the Middle Pleistocene Subseries/Subepoch and Chibanian Stage/Age (Fig. 14). The Chiba section is a segment of the Yoro River section which itself is one of five outcrops that comprise the CbCS (west to east): the Urajiro, Yanagawa, Yoro River, Yoro-Tabuchi, and Kokusabata sections (Table 1 of Haneda et al. 2020a; Suganuma et al. in press). A borehole, TB-2, near the Yoro-Tabuchi outcrop and 190 m northeast of the Chiba section (Hyodo et al. 2016, 2017) contributes to this composite section. Collectively, these sections span a distance of 7.4 km along strike and are stratigraphically linked by a series of tephra beds (Kazaoka et al. 2015). The GSSP is located at the base of the Byk-E tephra bed (Fig. 14d), a conspicuous regional marker 1 to 3 cm thick in the Chiba section (Nishida et al. 2016) that has been correlated with the YUT5 bed erupted from the Older Ontake volcano in the central part of Honshu approximately 250 km to the west (Takeshita et al. 2016).

### Geological background

The Chiba section exposes the middle of the Kokumoto Formation which itself is within the middle part of the Kazusa Group. The Kazusa Group is approximately 3000 m in thickness and represents the Lower and Middle Pleistocene infill of a forearc basin, the Kanto Tectonic Basin (Fig. 14b), resulting from the west–northwestward subduction of the Pacific plate beneath the Eurasian plate at the Izu–Bonin trench (Ito and Katsura 1992). Uplift began about one million years ago, resulting in the deeply incised gorges that characterize the Boso Peninsula. Interbedded sandstones and siltstones dominate the lithology, with the sandstones typically being turbiditic. The depositional environment has been variously interpreted as basin plain, lower fan and base of slope in the lower part of the Kazusa Group (Fig. 14b) but gradually shallowing upwards to upper slope and shelf environments at the top (Kazaoka et al. 2015). Deep-water massive sandstones in the middle and upper parts of the Kazusa Group represent hyperpycnal and sediment gravity flows originating from shelf-margin deltas or fan deltas. These flows were activated during the falling and lowstand stages of sea-level oscillations controlled primarily by glacioeustacy (Takao et al. 2020).

The Kokumoto Formation is approximately 350 m thick along the Yoro River and represents MIS 21–18 (~ 860–720 ka). It comprises thick silty beds along with alternating sand and thinner silt beds (Kazaoka et al. 2015). Where exposed along the CbCS, it is a muddy unit deposited from suspension under stable and calm bottom-water conditions, with sedimentary structures and trace fossil assemblages together indicating a continental slope setting (Nishida et al. 2016). In particular, the presence of the ichnogenus *Zoophycos* in the CbCS (Nishida et al. 2016) implies a water depth of more than 800–1000 m based on its modern bathymetric distribution (fig. 4 in Löwemark and Werner 2001), as noted by Izumi et al. (2021). Parallel bedding observed at the CbCS (Fig. 14c) attests to continuous deposition without slumping. This muddy unit, underlain and overlain by deep-water massive sandstones, is a relatively condensed section formed in the uppermost part of a transgressive systems tract (Takao et al. 2020) on the continental slope.

### Modern oceanography and climate

**Fig. 15:**
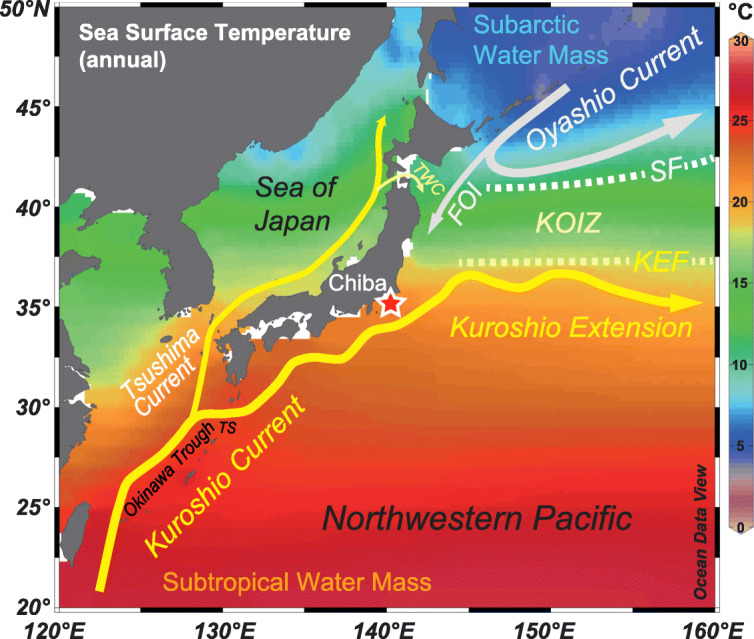
Present oceanography of the northwestern Pacific Ocean, showing annual sea-surface temperature from the World Ocean Atlas 2013 (Locarnini et al. 2013) drawn using Ocean Data View software (Schlitzer 2015). Major oceanographic features are included. TWC = Tsugaru Warm Current. SF = Subarctic Front formed by the Subarctic Current, an eastward extension of the Oyashio Current. KEF = Kuroshio Extension Front. KOIZ = Kuroshio–Oyashio interfrontal zone, a zone of mixing between the SF and KEF. TS = Tokara Strait. FOI = first Oyashio intrusion. Adapted from fig. 1 of Suganuma et al. (2018)

The area off the CbCS today experiences the highest oceanographic gradients in the western Pacific owing to the confluence of two major western boundary currents: the warm, nutrient-deficient north-flowing Kuroshio Current and the cold south-flowing nutrient-rich and less saline Oyashio Current. After converging, the Kuroshio Current flows eastwards as a jet known as the Kuroshio Extension along a frontal zone that separates the North Pacific Subpolar Gyre from the North Pacific Subtropical Gyre. This frontal system consists of the Subarctic Front to the north and Kuroshio Extension Front to the south, forming an intervening zone called the Kuroshio–Oyashio Interfrontal Zone (KOIZ) (Komatsu and Hiroe, 2019; Fig. 15). This area is therefore highly sensitive to changes in the strength and latitudinal position of the Kuroshio Extension which itself is driven by North Pacific, East Asian, and global climate oscillations on seasonal, decadal, and orbital time scales. The CbCS then is well positioned to record the evolving behaviour of this major oceanographic boundary system throughout MIS 20–18 (Suganuma et al. 2018; Haneda et al. 2020b; Kameo et al. 2020; Izumi et al. (2021), Balota et al. 2021; Kubota et al. 2021).

**Fig. 16:**
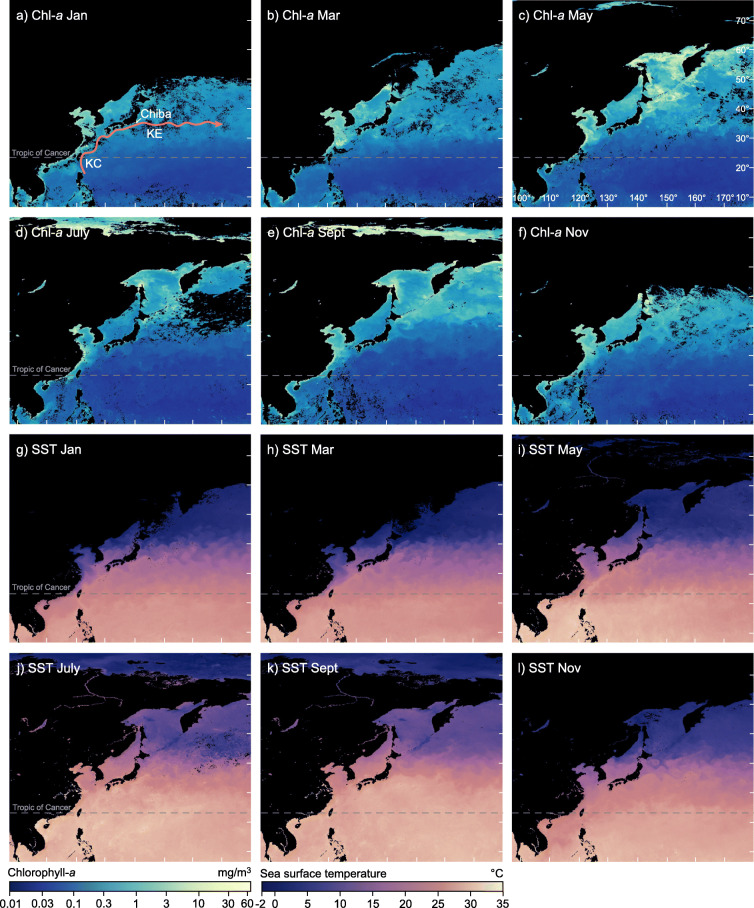
Monthly chlorophyll-*a* (Chl-*a*) concentrations and monthly sea-surface temperature (SST) for the western North Pacific in 2019 based on satellite observations. **a**–**f** Chlorophyll-*a* concentrations as a measure of primary (phytoplankton) productivity. The generalized positions of the Kuroshio Current (KC) and Kuroshio Extension (KE) are given in **a**. The position of the Kuroshio Extension Front (KEF; Fig. 15) is evident from sharply enhanced productivity around and north of ~ 30° N; greatest productivity in the mid-latitudes east and north of Chiba is during the boreal spring (March–May) with a subdued rise in the autumn (September–October). **g–l** Sea-surface temperature; the KEF moves only slightly northwards during summer–autumn but the latitudinal gradient across the KEF lessens significantly in summer with considerable northward diffusion of warm water during July–September. Note mesoscale eddies pinching off along the KEF and advecting heat northwards. NASA Earth Observations (2020)

Monthly satellite imagery of the western North Pacific for 2019 shows the position of the Kuroshio Extension Front and pronounced seasonal changes affecting sea-surface temperature and primary productivity (Fig. 16). Although the latitudinal position and flow speed of the Kuroshio Extension change little through the year, the magnitude of the Kuroshio Extension Front strength, as measured by the horizontal temperature gradient, is greatest during the cold season and least during the warm season (Chen 2008, Kida et al. 2015, Wang et al. 2016, Yu et al. 2020). Mesoscale perturbations along the Kuroshio Extension Front are also greater in winter (Wei et al. 2017). Seasonal variation in frontal strength is greatest off Japan and hence relevant to the CbCS. The Oyashio Current as observed along the continental slope off Hokkaido flows more weakly in summer and autumn, its total volume transport reaching 20–30 Sv in winter and spring but only 3–4 Sv in summer and autumn (Qiu 2019). As a result of these seasonal variations, the mixed layer in the Kuroshio region is deep (> 100 m) during winter, promoting the supply of nutrients to the surface, whereas in summer it is shallower (< 15 m) and nutrient depleted (Komatsu and Hiroe 2019). These seasonal differences are significant for the interpretation of climate proxies in the CbCS.

The Kuroshio Extension Front varies significantly at interannual to decadal frequencies with respect to strength, latitude, and elongated versus convoluted pathway (Wang et al. 2016; Yu et al. 2020), and its annual mean position off Japan has shifted between 33° and 37° N over the period 1993 to 2013 (Wang et al. 2016). This variability strongly correlates with the North Pacific Oscillation, a north–south seesaw between the Aleutian Low and the Pacific High below it (Fig. 4). The Aleutian Low intensifies during its positive phase and shifts northwards (Sugimoto and Hanawa 2009; Yu et al. 2020), favouring a strengthened and northward-moving Kuroshio Extension Front. Changes in the latitude of the Aleutian Low, a cold-season phenomenon that dissipates almost entirely in summer, may therefore be linked to the position of the Kuroshio Extension Front during MIS 19.

The Siberian High, North Pacific Oscillation, Arctic Oscillation, and North Atlantic Oscillation are major interlinked sea-level pressure systems in the northern hemisphere. The Arctic Oscillation (Thompson and Wallace 1998) is a large-scale surface-pressure system linked to the stratospheric polar vortex and alternating between a negative and positive mode. In its positive mode, surface pressure is high in the polar region which causes the encircling mid-latitude jet stream to intensify and confine cold air within this region. In negative mode, the surface pressure falls and the resulting zonal winds become weaker and more distorted, allowing cold arctic air masses to flow into the mid-latitudes. A coupling between the Siberian High and Aleutian Low has been proposed by Huang et al. (2016) and Kumar et al. (2019). Climate modelling experiments have also revealed linkages between the EAWM, which reflects the intensity of the Siberian High, and both the North Pacific Oscillation and Arctic Oscillation (Miao et al. 2020). Moreover, the coupling strength between the Arctic Oscillation and the EAWM is enhanced by increased ice cover over the East Siberian Seas (Wie et al. 2019). The Arctic Oscillation is also strongly linked to the North Atlantic Oscillation comprising the Azores High and the Iceland Low (Hamouda et al. 2021; Fig. 4). The connections between these major systems are not straightforward and may vary with global temperatures (Hamouda et al. 2021). Nonetheless, the Arctic Oscillation represents a plausible high-latitude link between North Atlantic and North Pacific climate processes during MIS 19 especially at times of increased ice cover over Siberia. These high-latitude linkages, along with the modelled (e.g. Moreno-Chamarro et al. 2020) and historical (Chen et al. 2019, Liu et al. 2020) relationship between the North Atlantic Oscillation, AMOC and position of the ITCZ, illustrate the tightly integrated nature of the global climate system.

### Chiba paleoceanography and paleoclimate through MIS 19

The CbCS has sedimentation rates of ~ 89 cm/ky across the GSSP (Suganuma et al. in press) and represents one of the most intensely researched intervals available to understand the climatic development of MIS 19. A benthic and planktonic foraminiferal isotope record at high stratigraphic resolution (Haneda et al. 2020b) has a structure remarkably similar to that of North Atlantic and Mediterranean records throughout MIS 19. Similarities include power spectra containing the harmonics of precession which implies low-latitude forcing throughout, and a gradual trend to higher benthic foraminiferal δ^18^O values in the latter part of MIS 19c that reflects a progressive increase in global ice volume. At the CbCS, a sharp increase in benthic and especially planktonic foraminiferal δ^18^O values marks the onset of MIS 19b and is taken to reflect the inception of glaciation. As with some Mediterranean records, MIS 19a is represented by four interstadials, i1–i4, and by four corresponding benthic marine isotope oscillations (MIS 19a-*o*1–4) (Fig. 11g, h). Haneda et al. (2020b) invoked shifts in the ITCZ to explain the coherence between the Chiba and Mediterranean records, as noted above.

**Fig. 17:**
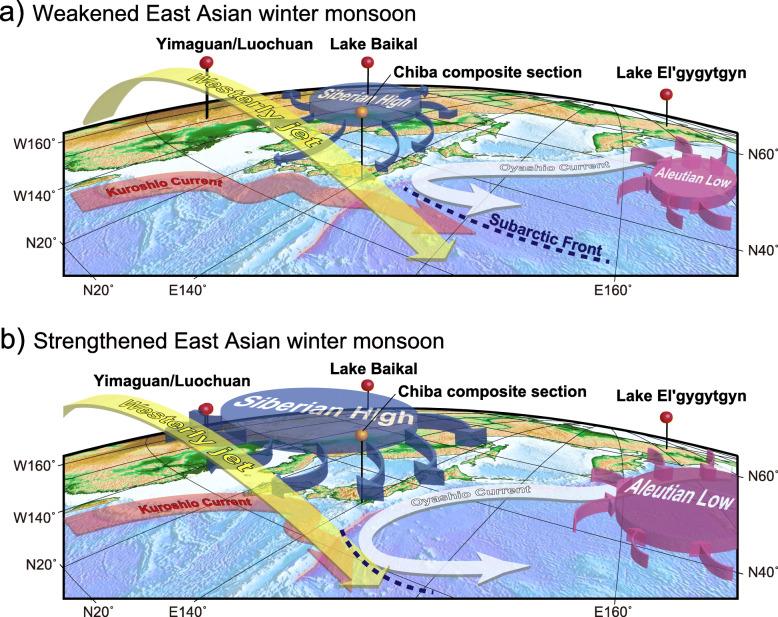
A schematic representation of the northwest Pacific showing the north/south migration of the westerly jet in winter and associated shifts of the Subarctic Front. A weaker east Asian winter monsoon (EAWM) (**a**) causes the Subarctic Front to move northwards (interstadial conditions), and a stronger EAWM (**b**) deflects it southwards (stadials). The EAWM is controlled by the thermal contrast between the Siberian High and Aleutian Low atmospheric pressure systems: when both are intensified (**b**), the EAWM is strongest. Locations of sites discussed in the text are shown. Adapted from fig. 15 of Suganuma et al. (2018)

The CbCS documents changes in the Kuroshio Extension system through MIS 19, including rapid north–south shifts associated with stadial/interstadial alternations during MIS 19b–a (Haneda et al. 2020b). Foraminiferal isotopes and other proxies reflect both warming and increased surface and near-surface water-column stratification during the interstadials (Fig. 11d–f). Because these proxies represent winter oceanographic conditions, they imply weaker mixing of the water column during winter. The East Asian Winter Monsoon (Fig. 4) largely controls winter wind strength today and was therefore likely weaker during the warmer intervals of MIS 19a (Suganuma et al. 2018). The intensity of the East Asian Winter Monsoon system is driven by the thermal contrast between the Siberian High (cold) and Aleutian Low (warm) pressure systems, both of which develop primarily during winter. A weak EAWM occurs when winter conditions in Siberia are relatively warm (low pressure) and those over the northwestern Pacific Ocean are relatively cold. A weak EAWM during the latter part of MIS 19 is supported by evidence from the Chinese loess record and has been attributed to a weak minimum in summer insolation at 65° N resulting in reduced ice accumulation over Siberia (Hao et al. 2012; Fig. 12e). Suganuma et al. (2018) proposed that under ice-free conditions, enhanced winter insolation at 50° N might also have contributed to a weak Siberian High, and consequently a weak EAWM in the low-eccentricity configuration of MIS 19. A weak EAWM will then have caused the position of the westerly jet to advance northwards. The latitudinal position of the Kuroshio Extension system is strongly influenced by that of the westerly jet and will have shifted northwards accordingly. During interstadials, therefore, the Kuroshio Extension system would have migrated northwards, bringing warm, stratified water masses to the CbCS (fig. 15 in Suganuma et al. 2018; Fig. 17).

While this mechanism explains the continued presence of warm, stratified waters at the CbCS late in MIS 19, it does not address the numerous abrupt stadial–interstadial alternations that characterize MIS 19b–a at this site or the similarity with North Atlantic–Mediterranean records, and it does not account for potential variation in the intensity of the Aleutian Low.

The loess records of China show a continually weak EAWM and EASM through the latter half of MIS 19 (Hao et al. 2012; Peng et al. 2020) and while this provides evidence for the persistence of unusually warm conditions late in MIS 19, the millennial-scale stadial–interstadial alternations that characterize MIS 19b–a are not clearly expressed. A combination of pedogenic processes and relatively low sedimentation rates likely accounts for this absence of abrupt changes. This itself introduces uncertainty into age models for the latter part of MIS 19 and hinders insights into the mechanisms driving stadial–interstadial alternations. The S7 palaeosol, now widely accepted as equating with MIS 19, has since been studied in detail by Zhang et al. (2020) who examined successions on the Chinese Loess Plateau for their mollusc content. They determined the earlier part of MIS 19 to be slightly warmer than at present, and the later part similar to present but with stronger climatic variability. In the east, warm conditions continued ~ 15 kyr into MIS 18, suggesting a relatively strong summer monsoon through MIS 19 and well into MIS 18, contrary to Hao et al. (2012, suppl. fig. 12) who showed progressive weakening through MIS 19. Again, however, clear coherent oscillations in the latter part of MIS 19 are not apparent.

The CbCS planktonic foraminiferal isotope data through MIS 19b–a (Haneda et al. 2020b) show four pronounced stadials (MIS 19-s1 to s4) and interstadials (MIS 19-i1 to i4) (Fig. 11). Less than 1000 years after the beginning of MIS 19b, the onset of stadial s1 is identified by a rapid and significant increase in planktonic δ^18^O values accompanied by increases in dinoflagellate cyst concentrations and especially a sharp (within ~ 300 years) increase in the relative abundance of the dinoflagellate *Protoceratium reticulatum* (Balota et al. 2021; Fig. 11). The dinoflagellate cyst record also shows a rapid transition out of stadial s1 as represented by a decrease in *Protoceratium reticulatum*. If this interval of abundance (biozone Df7 in Balota et al. 2021; Fig. 11c) indeed reflects the true duration of stadial s1, then at 2.48 kyr (772.85–770.37 ka) it is longer than the 1.0 and 1.2 kyr durations at Piànico-Sèllere and Montalbano Jonico, respectively (Nomade et al. 2019). The abrupt rise in *Protoceratium reticulatum* coincides with a sharp shift to heavier planktonic foraminiferal δ^18^O values, reasonably marking the onset of stadial s1, but this species remains abundant nearly half-way into the following interstadial i1. Indeed, its abrupt decline coincides with the sharp change to heavier benthic δ^18^O values marking the end of benthic isotope oscillation *o*1 (Fig. 11). The abundance of *Protoceratium reticulatum* appears to indicate the influence of cooler, mixed, nutrient-rich waters of the Kuroshio–Oyashio Interfrontal Zone resulting from a southward shift of the Kuroshio Extension (Balota et al. 2021; Fig. 15). Future ultra-high-resolution multiproxy studies are needed to fully understand these rapid climate changes and their respective planktonic and benthic expressions through MIS 19b–a. In any case, these stadials in the Chiba record appear to correspond well with cool/dry stadials in the Mediterranean and with intervals in the North Atlantic record characterized by IRD, meltwater, cooling, and reduced ventilation, all indicative of AMOC disruptions. This suggests a teleconnection between the North Atlantic and the western Pacific at this time.

Haneda et al. (2020b) used very high-resolution benthic and planktonic foraminiferal δ^18^O analyses of the CbCS throughout MIS 19 to estimate water-column stratification by comparing δ^18^O values between benthic foraminifera, the deep-dwelling (~ 300–400 m depth in the subtropical North Pacific) *Globorotalia inflata*, and the surface-dwelling *Globigerina bulloides*. While all records show millennial-scale alternations during MIS 19b–a, the amplitude of the *G. bulloides* record reasonably suggests that the source of these alternations was from surface-water processes (Haneda et al. 2020b). However, the onset of benthic isotope oscillation *o*2 appears to lead slightly that of interstadial i2 (Fig. 11g). Detailed multiproxy studies at ultra-high stratigraphic resolution are needed to resolve these small discrepancies. Variations in δ^18^O values have been considered primarily to reflect temperature, with Δ*T* (δ^18^O_benthic_ minus δ^18^O_bulloides_; Fig. 11e) representing the difference between bottom and surface water temperature (Haneda et al. 2020b, 2020c). The difference between δ^18^O values for *Globorotalia inflata* and *Globigerina bulloides* (Suganuma et al. 2018; fig. 2d of Kubota et al. 2021; Fig. 11f) and the relative abundance of the calcareous nannofossil *Florisphaera profunda* (Kameo et al. 2020; Fig. 11d) provide additional measures of surface and near-surface water-column stratification. Haneda et al. (2020b) showed that increasing stratification occurred during interstadials and vice versa for stadials, supporting the notion that stadial–interstadial oscillations represent latitudinal displacements of the Kuroshio Extension Front at the CbCS. Total organic carbon (TOC) values generally mirror the stadial–interstadial alternations of the latter part of MIS 19a, with increased values coinciding with interstadials i2, i3, and i4 (Izumi et al. 2021; Fig. 11b). Izumi et al. (2021) tentatively proposed that peaks at interstadials i2 and i3 represent enhanced organic matter preservation owing to water-column stratification rather than increased surface water productivity. This follows the reasoning that surface water productivity should be lower during interstadials as they mark the northward shift of the Kuroshio Current which is relatively poor in nutrients. Enhanced organic matter preservation is supported by geochemical data and evidence from trace fossils that indicate reduced oxygen levels in the bottom waters. However, among the highest values of TOC occur within stadial s1 which was influenced by the nutrient-rich Oyashio Current, as evidenced by the dinoflagellate cyst record (Balota et al. 2021; Fig. 11c). Izumi et al. (2021) acknowledged that more research is needed to understand the important relationship between surface water productivity, organic matter preservation, and stadial–interstadial alternations.

Latitudinal changes in the ITCZ best explain the millennial-scale oscillations at the CbCS and their teleconnection to the North Atlantic and Mediterranean records (Haneda et al. 2020b; Fig. 4). Disruptions to AMOC by meltwater release into the North Atlantic, and consequent triggering of the thermal bipolar seesaw, creates a strong thermal contrast between the northern and southern hemispheres. This causes the ITCZ to move southwards and the trade winds to intensify in association with a deepened Aleutian Low. The mid-latitude prevailing westerlies move in parallel and the consequent atmospheric reorganization causes the Kuroshio Extension also to shift southwards during stadials. Using a combination of Antarctic ice-core records and climate modelling over the past 720 kyr, Kawamura et al. (2017) showed that Antarctic warming events, linked to activation of the thermal bipolar seesaw, are most frequent when the climate is intermediate between glacial and interglacial states, as occurs at the beginning of MIS 19 (the Younger Dryas-type interruption of Termination IX) and the stadial–interstadial states of MIS 19b–a. These instabilities recorded at the CbCS and elsewhere therefore appear to reflect an intrinsic oscillation within the Earth system amplified during an intermediate glacial–interglacial state. Modelling results suggests that reduced CO_2_ concentrations along with extended Northern Hemisphere ice sheets, as must have been developing after the glacial inception near the end of MIS 19c (see above, also Vavrus et al. 2018), are prerequisites for such instability (Kawamura et al. 2017). To understand the rapid warming from stadial to interstadial states during MIS 19a, it may be relevant to examine the similarly rapid transition from the Heinrich 1 stadial to the Bølling-Allerød interstadial during the last deglaciation. Modelling studies show that gradually changing conditions can lead to an abrupt recovery of the AMOC and consequent rapid warming that marks the onset of the Bølling-Allerød interstadial (Obase and Abe-Ouchi 2019).

Time series analyses of planktonic foraminiferal δ^18^O records (Fig. 11g) including records of water-column stratification (Δ*T*; Fig. 11e) for the ChCS have revealed periodicities of a half-precession cycle throughout MIS 19 but also higher frequencies including those of the fourth harmonic of precession for MIS 19b–a (Haneda et al. 2020b). These harmonics of precession in the time series are derived from equatorial insolation (Fig. 11a). The influence of precession is strong at Lake Baikal at 51°–53° N, although it declines with increasing latitude (Prokopenko et al. 2006). The half-precession cycle at the CbCS is strongest within MIS 19c presumably owing to the absence of major ice melting events and AMOC disruption in the North Atlantic at this time. These high-latitude North Atlantic events then account for the stadial–interstadial alternations of MIS 19b–a via latitudinal displacements of the ITCZ, although paced by low-latitude insolation variations (Haneda et al. 2020b).

Despite the significant advances reviewed above (Suganuma et al. 2021), several aspects of the CbCS paleoenvironmental record remain incompletely known. Most proxies available at high stratigraphic resolution, including the foraminiferal isotopes, reflect the cooler seasons (Suganuma et al. 2018; Haneda et al. 2020b). Summer sea-surface temperatures and hence seasonal contrasts through MIS 19 are poorly understood but were presumably considerable then as they are now (Fig. 16).

Over the western Pacific at present, a significant divergence exists between winter and summer positions of the ITCZ owing to summer heating across Asia. For example, the ITCZ occurs over northern India in summer but shifts to just north of Australia in winter (Fig. 4) along with its associated trade winds. However, northern hemisphere ice sheets will have depressed this divergence and reduced the northward shift of the ITCZ in summer (Chiang and Friedman 2012; Schneider et al. 2014). In the CbCS, sea-surface temperature and near-surface stratification proxies are based on calcareous microfossils that accumulate calcite during winter (Suganuma et al. 2018; Haneda et al. 2020b). These will have differed substantially from proxies representing the summer months, although both will have been affected by the bipolar thermal seesaw. More research is needed to characterize summer land and ocean temperatures in the CbCS, especially during MIS 19b–a.

The dinoflagellate cyst record may represent in part a late spring–early autumn signal and shows significant fluctuations through MIS 19c and across stadial s1 into interstadial i1 within MIS 19a (zones Df7 and Df8 in Balota et al. 2021; Fig. 11c). These indicate an abrupt southward shift of the Kuroshio extension during glacial inception (stadial s1) and a similarly abrupt northward shift within interstadial i1 (biozone Df7 of Balota et al. 2021). For the latter part of MIS 19, it would then appear that the dynamics of the westerly jet and ITCZ are also reflected by warm-season as well as cold-season proxies. While this is to be expected, the remaining alternations of MIS 19a have yet to be analysed in detail for indicators of warm-season sea-surface conditions, and therefore variations in seasonality are not known.

A high-resolution pollen record at the CbCS similarly extends only to the MIS 19b–a boundary (Suganuma et al. 2018), although the terrestrial vegetation will have been sensitive to changes in spring–summer warmth and precipitation as well as winter frost during the stadial–interstadial oscillations of MIS 19a. An extension of this high-resolution pollen record through MIS 19a should yield information about variations in strength of the EASM across stadial–interstadial alternations. Indeed, many proxy records lack the ultra-high stratigraphic resolution needed to fully characterize changes through MIS 19b–a, although abundant opportunities are afforded by the high sedimentation rates (> 89 cm/kyr, Suganuma et al. 2018) in this part of the succession.

Further evidence for climatic oscillations in the latter part of MIS 19 during summer is found in the higher-latitude biogenic silica records of Lake Baikal which was strongly influenced by precessional variation (Prokopenko et al. 2006) and Si/Ti records of Lake El’gygytgyn (Wennrich et al. 2014) which seem to represent direct high-latitude teleconnections with the northern North Atlantic. These records reflect warm-season productivity and show pronounced interstadials i1 and i2 (Fig. 12c, d).

## Summary and conclusions

The GSSP defining the base of the Chibanian Stage and Middle Pleistocene Subseries at the Chiba section, Japan, was ratified on January 17, 2020, by the Executive Committee of the IUGS (Suganuma et al. in press). The GSSP occurs immediately below the top of MIS 19c and has an astronomical age of 774.1 ± 5.0 ka. The M–B reversal, with a directional midpoint just 1.1 m above the GSSP, an astronomical age of 772.9 ± 5.4 ka, and a duration of up to ~ 2.0 kyr, serves as the primary guide to the boundary. The two other candidate sections, the Ideale section at Montalbano Jonico and the Valle di Manche, both in Italy, were deemed to have equivocal (Valle di Manche) or imprecise (Montalbano Jonico) reversal records (Head 2019). This finalizes a process initiated by INQUA in 1973 to define the base of the Middle Pleistocene, a term in use since at least 1869. Although the Chibanian Stage is presently concurrent with the Middle Pleistocene Subseries, the introduction of a second stage for the Middle Pleistocene Subseries with its base near the onset of the mid-Brunhes event (MIS 12–11 transition, ~ 424 ka; Fig. 2) should be considered.

The M–B reversal facilitates global recognition in marine, terrestrial, and ice-core records, and places the GSSP appropriately in the middle of the Early–Middle Pleistocene Transition, an interval of profound and lasting climatic change (Head and Gibbard 2015b). MIS 19 was first labelled by Shackleton and Opdyke (1973; Fig. 6) who confirmed an earlier association of this interglacial stage with the M–B reversal (Hays et al. 1969; Fig. 5). Bassinot et al. (1994) were the first to subdivide MIS 19 formally, labelling events MIS 19.1, 19.2, and 19.3 (Fig. 8b). Tzedakis et al. (2012a, 2012b) were apparently the first to subdivide MIS 19 into lettered substages, MIS 19a, 19b, and 19c (Fig. 8c). Most subsequent authors have followed this three-lettered scheme but the boundary between MIS 19b and 19a has been applied inconsistently. The approach here is to restrict MIS 19b to the first interval of high foraminiferal isotopic values following MIS 19c (Fig. 7) following Nomade et al. (2019). The fine-scale subdivision of MIS 19 has been treated in various ways. However, climatostratigraphic units based potentially on multiple paleoenvironmental criteria, for which stadial-interstadial terminology is appropriate, are conceptually different from the benthic isotope signal which includes a global ice-volume component. For the latter part of MIS 19, it is proposed that stadial-interstadial labelling MIS 19-s1 to -s4 and MIS 19-i1 to -i4 be used independently of substages, with the three or four peaks in the benthic isotope record of MIS 19a separately labelled as benthic isotope oscillations MIS 19a-*o*1 to -*o*4 (Fig. 7).

MIS 19 is characterized by a reduced-amplitude 400 ky eccentricity cycle similar to our present interglacial (Fig. 2) but obliquity increased less rapidly and to a lower amplitude, and peak temperatures seem to have been generally lower than for the pre-industrial Holocene. CO_2_ levels seem to have been similar until about 8000 years ago when they began to rise in the Middle Holocene (Fig. 13). A Younger Dryas-like oscillation interrupts the deglaciation of Termination IX at several sites, possibly triggered by a brief AMOC disruption under these unusual orbital conditions (Haneda et al. 2020b; Marino et al. 2020). The onset of MIS 19 was driven by a steep rise in June insolation at 65° N with maximum obliquity in phase with minimum precession. MIS 19c extends from 790 to 785 ka to the expansion of ice sheets (glacial inception) at 774–777 ka, the timing influenced by the time scales used, and spans full interglacial conditions which lasted for around 10 to 12.5 kyr. Records (Table 2) confirm the brevity of full interglacial conditions during MIS 19 compared with most later interglacials, including MIS 11 which has a similar orbital configuration, and results from an unusually early glacial inception relative to the obliquity cycle (Tzedakis et al. 2012a, their fig. 6). Any comparisons with later interglacials should consider not just orbital configuration but also the causes and effects of increased quasi-100 kyr periodicity during and after the Early–Middle Pleistocene transition (Head and Gibbard 2015b).

During MIS 19c, both Pacific and North Atlantic–Mediterranean planktonic records show variability in the half- or quarter-precession bandwidth, indicating the influence of equatorial insolation variation at low and mid-latitudes at a time when AMOC disruption was minimal. The interruption of westerlies carrying moisture to the western Mediterranean may have led to the northward transport of moisture, feeding ice sheets which were progressively expanding through much of MIS 19c as boreal summer insolation decreased (Sánchez-Goñi et al. 2016). The inception of glaciation (774–777 ka) at the end of MIS 19c presents a cluster of climatostratigraphic signals that can assist in identifying the Early–Middle Pleistocene boundary (774.1 ka) globally.

MIS 19b–a corresponds to a second precessional minimum, in antiphase with obliquity, that results in a suppressed insolation peak (Fig. 13) transposed onto a longer-term trend of increasing global ice volume. Global ice accumulation during the latter part of MIS 19c appears to have crossed a climate threshold, with MIS 19b marking the first of three or four AMOC disruptions triggered by ice-calving and freshwater release into the northern North Atlantic, and indicated by ice-rafted debris and sortable silt records at ODP Site 983 and elsewhere. These AMOC disruptions led to activation of the thermal bipolar seesaw, explaining a slightly lagged phase relationship with three AIMs (warming events) in the Antarctic ice-core record (Tzedakis et al. 2012b; Fig. 8c). As a result of these oscillations, three or four interstadials with characteristically abrupt transitions are represented during MIS 19b–a, both in the Asian–Pacific and North Atlantic–Mediterranean realms. The coherence of these oscillations on a global scale is best explained by shifts in the ITCZ (Fig. 4) as a result of thermal contrast between northern and southern hemispheres created by the bipolar seesaw (Chiang and Friedman 2012; Schneider et al. 2014; Nomade et al. 2019; Haneda et al. 2020b). For example, a warming of the southern hemisphere would displace the ITCZ southwards along with the mid-latitude westerlies. This in turn would have shifted the Subarctic Front southwards, initiating stadial conditions in the northern hemisphere. Stadial–interstadial oscillations occur at or close to the harmonics of precession, and their transition from one state to the other may take less than 200 years. These AMOC-triggered oscillations may therefore have been paced by equatorial insolation forcing, even at a time when the effects of precession were subdued (Ferretti et al. 2015), and in some regions amplified by the monsoon system (Nomade et al. 2019; Haneda et al. 2020b).

Although MIS 19c represents a close orbital analogue to the present interglacial, the dominant ~ 5 kyr cyclicity in global records of MIS 19 contrasts with the dominant 2.5 kyr cyclicity of MIS 1, calling into question the assumed close similarity of these two interglacial stages (Sánchez-Goñi et al. 2016). The precise mechanism driving AMOC events in the northern North Atlantic particularly during MIS 19b–a, and the teleconnections linking these events with stadial–interstadial oscillations in the eastern Siberian El-gygytgyn record (Fig. 12c), is not known. The Siberian High, North Pacific Oscillation, Arctic Oscillation, and North Atlantic Oscillation, representing the northern hemisphere’s major atmospheric pressure systems, appear to be interlinked over historical time scales and may have provided high-latitude teleconnections during MIS 19. Additional high-resolution studies are needed to explore these potential connections during MIS 19. Detailed marine records of MIS 19 are conspicuously missing from the southern hemisphere (Fig. 4) yet such information is needed to test hypotheses involving interhemispheric processes.

## Data Availability

Not applicable

## Abbreviations

AIM: Antarctic Isotope Maximum
AMOC: Atlantic meridional overturning circulation
CbCS: Chiba composite section
EPICA: European Project for Ice Coring in Antarctica
GSSP: Global Boundary Stratotype Section and Point
ICS: International Commission on Stratigraphy
IODP: Integrated Ocean Drilling Program
INQUA: International Union for Quaternary Research
IRD: Ice-rafted debris
ITCZ: Intertropical Convergence Zone
IUGS EC: Executive Committee of the International Union of Geological Sciences
M–B: Matuyama–Brunhes
MIS: Marine Isotope Stage
ODP: Ocean Drilling Program
SQS: ICS Subcommission on Quaternary Stratigraphy

## References

[CR1] Abomriga WM (2018). Central North Atlantic (IODP Site U1313) paleoceanography based on a high-resolution dinoflagellate cyst record across the Early–Middle Pleistocene boundary (Marine Isotope Stages 20–18, ~ 810 –741 ka).

[CR2] Anonymous (1988). Biostratigraphy rejected for Pleistocene subdivisions. Episodes.

[CR3] Arrhenius G (1952). Sediment cores from the East Pacific. Swedish Deep-Sea Exped (1947–1948) Repts.

[CR4] Azzarone M, Ferretti P, Rossi V, Scarponi D, Capraro L, Macrì P, Huntley JW, Faranda C (2018). Early-Middle Pleistocene benthic turnover and oxygen isotope stratigraphy from the Central Mediterranean (Valle di Manche, Crotone Basin, Italy): Data and trends. Data Brief.

[CR5] Balota EJ, Head MJ, Okada M, Suganuma Y, Haneda Y (2021) Paleoceanography and dinoflagellate cyst stratigraphy across the Lower–Middle Pleistocene Subseries (Calabrian–Chibanian Stage) boundary at the Chiba composite section, Japan. Prog Earth Planet Sci (this issue) 10.1186/s40645-021-00438-3 PMC8550375 34722118

[CR6] Barth AM, Clark PU, Bill NS, Feng H, Pisias NG (2018). Climate evolution across the Mid-Brunhes Transition. Clim Past.

[CR7] Bassinot FC, Labeyrie LD, Vincent E, Quidelleur X, Shackleton NJ, Lancelot Y (1994). The astronomical theory of climate and the age of the Brunhes-Matuyama magnetic reversal. Earth Planet Sci Lett.

[CR8] Bazin L, Landais A, Lemieux-Dudon B, Toyé Mahamadou Kele H, Veres D, Parrenin F, Martinerie P, Ritz C, Capron E, Lipenkov V, Loutre M-F, Raynaud D, Vinther B, Svensson A, Rasmussen SO, Severi M, Blunier T, Leuenberger M, Fischer H, Masson-Delmotte V, Chappellaz J, Wolff E (2013). An optimized multi-proxy, multi-site Antarctic ice and gas orbital chronology (AICC2012): 120–800 ka. Clim Past.

[CR9] Bereiter B, Eggleston S, Schmitt J, Nehrbass-Ahles C, Stocker TF, Fischer H, Kipfstuhl S, Chappellaz J (2015). Revision of the EPICA Dome C CO_2_ record from 800 to 600 kyr before present. Geophys Res Lett.

[CR10] Berger A, Loutre MF (1991). Insolation values for the climate of the last 10 million years. Quat Sci Rev.

[CR11] Berger A, Yin Q, Nifenecker H, Poitou J (2017). Slowdown of global surface air temperature increase and acceleration of ice melting. Earth’s Future.

[CR12] Berggren WA, Hilgen FJ, Langereis CG, Kent DV, Obradovich JD, Raffi I, Raymo ME, Shackleton NJ (1995). Late Neogene chronology: New perspectives in high-resolution stratigraphy. Geol Soc Am Bull.

[CR13] Bertini A, Toti F, Marino M, Ciaranfi N (2015). Vegetation and climate across the Early–Middle Pleistocene transition at the Montalbano Jonico section (southern Italy). Quat Int.

[CR14] Björck S, Walker MJC, Cwynar LC, Johnsen S, Knudsen K-L, Lowe JJ, Wohlfarth B, Members INTIMATE (1998). An event stratigraphy for the Last Termination in the North Atlantic region based on the Greenland ice-core record: a proposal by the INTIMATE group. J Quat Sci.

[CR15] Bosmans JHC, Hilgen FJ, Tuenter E, Lourens LJ (2015). Obliquity forcing of low-latitude climate. Clim Past.

[CR16] Bowen DQ (1988). Quaternary geology: a stratigraphic framework for multidisciplinary work.

[CR17] Butzer KW, Isaac GL (1975). After the Australopithecines.

[CR18] Capraro L, Asioli A, Backman J, Bertoldi R, Channell JET, Massari F, Rio D, Head MJ, Gibbard PL (2005). Climatic patterns revealed by pollen and oxygen isotope records across the Matuyama–Brunhes Boundary in the central Mediterranean (southern Italy). Early–Middle Pleistocene transitions: the land–ocean evidence: Geological Society of London, Special Publication 247.

[CR19] Capraro L, Ferretti P, Macrì P, Scarponi D, Fornaciari E, Xian F, Zhou W, Kong X, Boschi V (2018). The ^10^Be record as a proxy of paleomagnetic reversals and excursions: a Mediterranean perspective. Alpine Mediterr Q.

[CR20] Capraro L, Ferretti P, Macrì P, Scarponi D, Tateo F, Fornaciari E, Bellini G, Dalan G (2017). The Valle di Manche section (Calabria, Southern Italy): A high resolution record of the Early–Middle Pleistocene transition (MIS 21–MIS 19) in the Central Mediterranean. Quat Sci Rev.

[CR21] Capraro L, Rio D, Sprovieri R, Channell JET, Vai GB (2004). A candidate section for defining the Lower-Middle Pleistocene boundary.

[CR22] Capraro L, Tateo F, Ferretti P, Fornaciari E, Macrì P, Scarponi D, Preto N, Xian F, Kong X, Xie X (2019). A Mediterranean perspective on ^10^Be, sedimentation and climate around the Matuyama/Brunhes boundary: *les liaisons dangereuses*?. Quat Sci Rev.

[CR23] Channell JET (2017). Complexity in Matuyama–Brunhes polarity transitions from North Atlantic IODP/ODP deep-sea sites. Earth Planet Sci Lett.

[CR24] Channell JET, Hodell DA (2017). Comment on Mark et al. (2017): High-precision ^40^Ar/^39^Ar dating of Pleistocene tuffs and temporal anchoring of the Matuyama–Brunhes boundary. Quaternary Geochronology, 39, 1–23. Quat Geochronol.

[CR25] Channell JET, Hodell DA, Curtis JH (2016). Relative paleointensity (RPI) and oxygen isotope stratigraphy at IODP Site U1308: North Atlantic RPI stack for 1.2–2.2 Ma (NARPI-2200) and age of the Olduvai Subchron. Quat Sci Rev.

[CR26] Channell JET, Kleiven HF (2000). Geomagnetic palaeointensitites and astrochronological ages for the Matuyama–Brunhes boundary and the boundaries of the Jaramillo Subchron: Palaeomagnetic and oxygen isotope records from ODP Site 983. Phil Trans R Soc Lond Ser A.

[CR27] Channell JET, Singer BS, Jicha BR (2020). Timing of Quaternary geomagnetic reversals and excursions in volcanic and sedimentary archives. Quat Sci Rev.

[CR28] Chen H-F, Liu Y-C, Chiang C-W, Liu X, Chou Y-M, Pan H-J (2019). China’s historical record when searching for tropical cyclones corresponding to Intertropical Convergence Zone (ITCZ) shifts over the past 2 kyr. Clim Past.

[CR29] Chen S (2008). The Kuroshio Extension front from satellite sea surface temperature measurements. J Oceanogr.

[CR30] Cheng H, Sinha A, Wang X, Cruz FW, Edwards RL (2012). The Global Paleomonsoon as seen through speleothem records from Asia and the Americas. Clim Dyn.

[CR31] Chiang JCH, Friedman AR (2012). Extratropical cooling, interhemispheric thermal gradients, and tropical climate change. Annu Rev Earth Planet Sci.

[CR32] Ciaranfi N, Head MJ, Marino M (2015). Report of the Field Workshop on the Lower–Middle Pleistocene transition in Italy. Quat Perspect.

[CR33] Cita MB (2008). Summary of Italian marine stages of the Quaternary. Episodes.

[CR34] Cita MB, Capraro L, Ciaranfi N, Di Stefano E, Lirer F, Maiorano P, Marino M, Raffi I, Rio D, Sprovieri R, Stefanelli S, Vai GB (2008). The Calabrian Stage redefined. Episodes.

[CR35] Cita MB, Capraro L, Ciaranfi N, Di Stefano E, Marino M, Rio D, Sprovieri R, Vai GB (2006). Calabrian and Ionian: a proposal for the definition of Mediterranean stages for the Lower and Middle Pleistocene. Episodes.

[CR36] Cohen KM, Finney SC, Gibbard PL, Fan JX (2013, updated) The ICS International Chronostratigraphic Chart. Episodes 36:199–204. Version 2020/01. https://stratigraphy.org/icschart/ChronostratChart2020-01.pdf

[CR37] Cohen KM, Gibbard PL (2019). Global chronostratigraphical correlation table for the last 2.7 million years, version 2019 QI-500. Quat Int.

[CR38] Cox A, Doell RR, Dalrymple GB (1963). Geomagnetic polarity epochs and Pleistocene geochronometry. Nature.

[CR39] Cox A, Doell RR, Dalrymple GB (1964). Reversals of the Earth’s magnetic field. Science.

[CR40] Dawkins B (1878). On the evidence afforded by the caves of Great Britain as to the antiquity of Man. J Anthropol Inst G B Irel.

[CR41] Doell RR, Dalrymple GB (1966). Geomagnetic polarity epochs: a new polarity event and the age of the Brunhes–Matuyama boundary. Science.

[CR42] Elderfield H, Ferretti P, Greaves M, Crowhurst S, McCave IN, Hodell D, Piotrowski AM (2012). Evolution of ocean temperature and ice volume through the mid-Pleistocene climate transition. Science.

[CR43] Emanuele D, Ferretti P, Palumbo E, Amore FO (2015). Sea-surface dynamics and palaeoenvironmental changes in the North Atlantic Ocean (IODP Site U1313) during Marine Isotope Stage 19 inferred from coccolithophore assemblages. Palaeogeogr Palaeoclimatol Palaeoecol.

[CR44] Emeis K-C, Sakamoto T, Wehausen R, Brumsack H-J (2000). The sapropel record of the eastern Mediterranean Sea – results of Ocean Drilling Program Leg 160. Palaeogeogr Palaeoclimatol Palaeoecol.

[CR45] Emiliani C (1955). Pleistocene temperatures. J Geol.

[CR46] Emiliani C (1966). Paleotemperature analysis of Caribbean cores P6304-8 and P6304-9 and a generalized temperature curve for the past 425,000 years. J Geol.

[CR47] EPICA Community Members (2006). One-to-one coupling of glacial climate variability in Greenland and Antarctica. Nature.

[CR48] Evans ME, Muxworthy AR (2018). A re-appraisal of the proposed rapid Matuyama–Brunhes geomagnetic reversal in the Sulmona Basin, Italy. Geophys J Int.

[CR49] Ferretti P, Crowhurst SJ, Naafs BDA, Barbante C (2015). The marine isotope stage 19 in the mid-latitude North Atlantic Ocean: astronomical signature and intra-interglacial variability. Quat Sci Rev.

[CR50] Forbes E (1846). On the connexion between the distribution of the existing fauna and flora of the British Isles, and the geological changes which have affected their area, especially during the epoch of the Northern Drift. Mem Geol Surv Great Britain.

[CR51] Ganopolski A, Winkelmann R, Schellnhuber HJ (2016). Critical insolation–CO_2_ relation for diagnosing past and future glacial inception. Nature.

[CR52] Giaccio B, Castorina F, Nomade S, Scardia G, Voltaggio M, Sagnotti L (2013). Revised chronology of the Sulmona lacustrine succession, central Italy. J Quat Sci.

[CR53] Giaccio B, Regattieri E, Zanchetta G, Nomade S, Renne PR, Sprain CJ, Drysdale RN, Tzedakis PC, Messina P, Scardia G, Sposato A, Bassinot F (2015). Duration and dynamics of the best orbital analogue to the present interglacial. Geology.

[CR54] Glass B, Ericson DB, Heezen BC, Opdyke ND, Glass JA (1967). Geomagnetic reversals and Pleistocene chronology. Nature.

[CR55] Gradstein FM, Ogg JG, Smith AG (2005). A geologic time scale 2004.

[CR56] Hamouda ME, Pasquero C, Tziperman E (2021). Decoupling of the Arctic Oscillation and North Atlantic Oscillation in a warmer climate. Nat Clim Chang.

[CR57] Haneda Y, Okada M, Kubota Y, Suganuma Y (2020). Millennial-scale hydrographic changes in the northwestern Pacific during marine isotope stage 19: Teleconnections with ice melt in the North Atlantic. Earth Planet Sci Lett.

[CR58] Haneda Y, Okada M, Kubota Y, Suganuma Y (2020). Corrigendum to “Millennial-scale hydrographic changes in the northwestern Pacific during marine isotope stage 19: Teleconnections with ice melt in the North Atlantic”. Earth Planet. Sci. Lett. 531 (2020) 115936. Earth Planet Sci Lett.

[CR59] Haneda Y, Okada M, Suganuama Y, Kitamura T (2020). A full sequence of the Matuyama–Brunhes geomagnetic reversal in the Chiba composite section, central Japan. Prog Earth Planet Sci.

[CR60] Hao Q, Wang L, Oldfield F, Peng S, Qin L, Song Y, Xu B, Qiao Y, Bloemendal J, Guo Z (2012). Delayed build-up of Arctic ice sheets during 400,000-year minima in insolation variability. Nature.

[CR61] Harkness R (1869). IV.—On the Middle Pleistocene deposits. Geol Mag.

[CR62] Hays JD, Saito T, Opdyke ND, Burckle LH (1969). Pliocene-Pleistocene sediments of the Equatorial Pacific: their paleomagnetic, biostratigraphic, and climatic record. Geol Soc Am Bull.

[CR63] Head MJ (2019). Formal subdivision of the Quaternary System/Period: present status and future directions. Quat Int.

[CR64] Head MJ, Aubry M-P, Walker M, Miller KG, Pratt BR (2017). A case for formalizing subseries (subepochs) of the Cenozoic Era. Episodes.

[CR65] Head MJ, Gibbard PL, Head MJ, Gibbard PL (2005). Early–Middle Pleistocene transitions: an overview and recommendation for the defining boundary. Early–Middle Pleistocene transitions: the land–ocean evidence: Geological Society of London, Special Publication 247.

[CR66] Head MJ, Gibbard PL (2015). Formal subdivision of the Quaternary System/Period: past, present, and future. Quat Int.

[CR67] Head MJ, Gibbard PL (2015). Early–Middle Pleistocene transitions: linking terrestrial and marine realms. Quat Int.

[CR68] Head MJ, Pillans B, Farquhar SA (2008). The Early–Middle Pleistocene transition: characterization and proposed guide for the defining boundary. Episodes.

[CR69] Head MJ, Pillans B, Zalasiewicz JA (in press) Formal ratification of subseries for the Pleistocene Series of the Quaternary System. Episodes

[CR70] Huang W, Wang B, Wright JS, Chen R (2016). On the non-stationary relationship between the Siberian High and Arctic Oscillation. PLoS ONE.

[CR71] Hyodo M, Bradak B, Okada M, Katoh S, Kitaba I, Dettman DL, Hayashi H, Kumazawa K, Hirose K, Kazaoka O, Shikoku K, Kitamura A (2017). Millennial-scale northern Hemisphere Atlantic-Pacifc climate teleconnections in the earliest Middle Pleistocene. Sci Rep.

[CR72] Hyodo M, Katoh S, Kitamura A, Takasaki K, Matsushita H, Kitaba I, Tanaka I, Nara M, Matsuzaki T, Dettman DL, Okada M (2016). High resolution stratigraphy across the early–middle Pleistocene boundary from a core of the Kokumoto Formation at Tabuchi, Chiba prefecture, Japan. Quat Int.

[CR73] Ito M, Katsura Y (1992). Inferred glacio-eustatic control for high-frequency depositional sequences of the Plio-Pleistocene Kazusa Group, a forearc basin fill in Boso Peninsula, Japan. Sediment Geol.

[CR74] Izumi K, Haneda Y, Suganuma Y, Okada M, Kubota Y, Nishida N, Kawamata M, Matsuzaki T (2021). Multiproxy sedimentological and geochemical analyses across the Lower–Middle Pleistocene boundary: chemostratigraphy and paleoenvironment of the Chiba composite section, central Japan. Prog Earth Planet Sci.

[CR75] Jansen JHF, Kuijpers A, Troelstra SR (1986). A Mid-Brunhes climatic event: long-term changes in global atmosphere and ocean circulation. Science.

[CR76] Jouzel J, Masson-Delmotte V, Cattani O, Dreyfus G, Falourd S, Hoffmann G, Minster B, Nouet J, Barnola JM, Chappellaz J, Fischer H, Gallet JC, Johnsen S, Leuenberger M, Loulergue L, Luethi D, Oerter H, Parrenin F, Raisbeck G, Raynaud D, Schilt A, Schwander J, Selmo E, Souchez R, Spahni R, Stauffer B, Steffensen JP, Stenni B, Stocker TF, Tison JL, Werner M, Wolff EW (2007). Orbital and millennial Antarctic climate variability over the past 800,000 years. Science.

[CR77] Kameo K, Kubota Y, Haneda Y, Suganuma Y, Okada M (2020). Calcareous nannofossil biostratigraphy of the Lower–Middle Pleistocene boundary of the GSSP, Chiba composite section in the Kokumoto Formation, Kazusa Group, central Japan, and implications for sea-surface environmental changes. Prog Earth Planet Sci.

[CR78] Kawai N (1951). Magnetic polarization of Tertiary rocks in Japan. J Geophys Res.

[CR79] Kawamura K, Abe-Ouchi A, Motoyama H, Ageta Y, Aoki S, Azuma N, Fujii Y, Fujita K, Fujita S, Fukui K, Furukawa T, Furusaki A, Goto-Azuma K, Greve R, Hirabayashi M, Hondoh T, Hori A, Horikawa S, Horiuchi K, Igarashi M, Iizuka Y, Kameda T, Kanda H, Kohno M, Kuramoto T, Matsushi Y, Miyahara M, Miyake T, Miyamoto A, Nagashima Y, Nakayama Y, Nakazawa T, Nakazawa F, Nishio F, Obinata I, Ohgaito R, Oka A, Okuno J, Okuyama J, Oyabu I, Parrenin F, Pattyn F, Saito F, Saito T, Saito T, Sakurai T, Sasa K, Seddik H, Shibata Y, Shinbori K, Suzuki K, Suzuki T, Takahashi A, Takahashi K, Takahashi S, Takata M, Tanaka Y, Uemura R, Watanabe G, Watanabe O, Yamasaki T, Yokoyama K, Yoshimori M, Yoshimoto T (2017). State dependence of climatic instability over the past 720,000 years from Antarctic ice cores and climate modeling. Sci Adv.

[CR80] Kazaoka O, Suganuma Y, Okada M, Kameo K, Head MJ, Yoshida T, Kameyama S, Nirei H, Aida N, Kumai H (2015). Stratigraphy of the Kazusa Group, Central Japan: a high-resolution marine sedimentary sequence from the Lower to Middle Pleistocene. Quat Int.

[CR81] Kida S, Mitsudera H, Aoki S, Guo X, Ito S, Kobashi F, Komori N, Kubokawa A, Miyama T, Morie R, Nakamura H, Nakamura T, Nakano H, Nishigaki H, Nonaka M, Sasaki H, Sasaki YN, Suga T, Sugimoto S, Taguchi B, Takaya K, Tozuka T, Tsujino H, Usui N (2015). Oceanic fronts and jets around Japan: a review. J Oceanogr.

[CR82] Kleinen T, Mikolajewicz U, Brovkin V (2020). Terrestrial methane emissions from the Last Glacial Maximum to the preindustrial period. Clim Past.

[CR83] Kleiven HF, Hall IR, McCave IN, Knorr G, Jansen E (2011). Coupled deep-water flow and climate variability in the middle Pleistocene North Atlantic. Geology.

[CR84] Komatsu K, Hiroe Y (2019) Structure and impact of the Kuroshio nutrient stream. In: Nagai T, Saito H, Suzuki K, Takahashi M. (eds) Kuroshio Current: physical, biogeochemical, and ecosystem dynamics. Geophys Monogr 243:85–104

[CR85] Konijnendijk TYM, Ziegler M, Lourens LJ (2014). Chronological constraints on Pleistocene sapropel depositions from high-resolution geochemical records of ODP Sites 967 and 968. Newsl Stratigr.

[CR86] Kubota Y, Haneda Y, Kameo K, Itaki T, Hayashi H, Shikoku K, Izumi K, Head MJ, Suganuma Y, Okada M (2021) Paleoceanography of the northwestern Pacific across the Early–Middle Pleistocene boundary (Marine Isotope Stages 20–18). Prog Earth Planet Sci 8(29):1–24 10.1186/s40645-020-00395-3 PMC8550468 34722117

[CR87] Kumar A, Lo EYM, Switzer AD (2019). Relationship between East Asian cold surges and synoptic patterns: A new coupling framework. Climate.

[CR88] Laskar J, Fienga A, Gastineau M, Manche H (2011). La2010: a new orbital solution for the long-term motion of the Earth. Astron Astrophys.

[CR89] Laskar J, Joutel F, Boudin F (1993). Orbital, precessional and insolation quantities for the Earth from –20 Myr to + 10 Myr. Astron Astrophys.

[CR90] Laskar J, Robutel P, Joutel F, Gastineau M, Correia ACM, Levrard B (2004). A long-term numerical solution for the insolation quantities of the Earth. Astron Astrophys.

[CR91] Lisiecki LE (undated) MIS boundary ages from http://www.lorraine-lisiecki.com/LR04_MISboundaries.txt. Accessed 18 May 2020.

[CR92] Lisiecki LE, Raymo ME (2005). A Plio-Pleistocene stack of 57 globally distributed benthic δ^18^O records. Paleoceanography.

[CR93] Lisiecki LE, Raymo ME (2009). Diachronous benthic δ^18^O responses during late Pleistocene terminations. Paleoceanography.

[CR94] Lisiecki LE, Stern JV (2016). Regional and global benthic δ^18^O stacks for the last glacial cycle. Paleoceanography.

[CR95] Liu C, Liao X, Qiu J, Yang Y, Feng X, Allan RP, Cao N, Long J, Xu J (2020). Observed variability of intertropical convergence zone over 1998–2018. Environ Res Lett.

[CR96] Liu Y, Lo L, Shi Z, Wei K-Y, Chou C-J, Chen Y-C, Chuang C-K, Wu C-C, Mii H-S, Peng Z, Amakawa H, Burr GS, Lee S-Y, DeLong KL, Elderfield H, Shen C-C (2015). Obliquity pacing of the western Pacific Intertropical Convergence Zone over the past 282,000 years. Nat Commun.

[CR97] Locarnini RA, Mishonov AV, Antonov JI, Boyer TP, Garcia HE, Baranova OK, Zweng MM, Paver CR, Reagan JR, Johnson DR, Hamilton M, Seidov D (2013). World Ocean Atlas 2013, Volume 1: Temperature. Levitus S (ed), Mishonov A (technical ed) NOAA Atlas NESDIS 73.

[CR98] Lourens LJ (2004). Revised tuning of Ocean Drilling Program Site 964 and KC01B (Mediterranean) and implications for the δ^18^O, tephra, calcareous nannofossil, and geomagnetic reversal chronologies of the past 1.1 Myr. Paleoceanography.

[CR99] Löwemark L, Werner F (2001). Dating errors in high-resolution stratigraphy: a detailed X-ray radiograph and AMS-^14^C study of *Zoophycos* burrows. Mar Geol.

[CR100] Lyell C (1839). Éléments de géologie.

[CR101] Lyell C (1863). The geological evidences of the antiquity of Man.

[CR102] Lyell C (1865). Elements of geology.

[CR103] Lyell C (1873). The geological evidences of the antiquity of Man.

[CR104] Macrì P, Capraro L, Ferretti P, Scarponi D (2018). A high-resolution record of the Matuyama–Brunhes transition from the Mediterranean region: The Valle di Manche section (Calabria, Southern Italy). Phys Earth Planet Inter.

[CR105] Maiorano P, Bertini A, Capolongo D, Eramo G, Gallicchio S, Girone A, Pinto D, Toti F, Ventruti G, Marino M (2016). Climate signatures through Marine Isotope Stage 19 in the Montalbano Jonico section (Southern Italy): A land–sea perspective. Palaeogeogr Palaeoclimatol Palaeoecol.

[CR106] Mangerud J, Andersen ST, Berglund BE, Donner JJ (1974). Quaternary stratigraphy of Norden, a proposal for terminology and classification. Boreas.

[CR107] Marino M, Bertini A, Ciaranfi N, Aiello G, Barra D, Gallicchio S, Girone A, La Perna R, Lirer F, Maiorano P, Petrosino P, Toti F (2015). Paleoenvironmental and climatostratigraphic insights for Marine Isotope Stage 19 (Pleistocene) at the Montalbano Jonico succession, South Italy. Quat Int.

[CR108] Marino M, Girone A, Gallicchio S, Herbert T, Addante M, Bazzicalupo P, Quivelli O, Bassinot F, Bertini A, Nomade S, Ciaranfi N, Maiorano P (2020). Climate variability during MIS 20–18 as recorded by alkenone-SST and calcareous plankton in the Ionian Basin (central Mediterranean). Palaeogeogr Palaeoclimatol Palaeoecol.

[CR109] Mark DF, Renne PR, Dymock R, Smith VC, Simon JI, Morgan LE, Staff RA, Ellis BS (2017). High-precision ^40^Ar/^39^Ar dating of Pleistocene tuffs and temporal anchoring of the Matuyama-Brunhes boundary. Quat Geochronol.

[CR110] Matuyama M (1929). On the direction of magnetization of basalt in Japan, Tyosen and Manchuria. Proc Imperial Acad (Tokyo).

[CR111] Miao J, Wang T, Wang H (2020). Interdecadal variations of the East Asian winter monsoon in CMIP5 preindustrial simulations. J Clim.

[CR112] Moreno-Chamarro E, Marshall J, Delworth TL (2020). Linking ITCZ migrations to the AMOC and North Atlantic/Pacific SST decadal variability. J Clim.

[CR113] Moscariello A, Ravazzi C, Brauer A, Mangili C, Chiesa S, Rossi S, de Beaulieu JL, Reille M (2000). A long lacustrine record from the Piànico-Sèllere basin (Middle–Late Pleistocene, Northern Italy). Quat Int.

[CR114] NASA Earth Observations (2020). NEO: NASA Earth Observations. https://neo.sci.gsfc.nasa.gov

[CR115] Ninkovich D, Opdyke N, Heezen BC, Foster JH (1966). Paleomagnetic stratigraphy, rates of deposition and tephrachronology in North Pacific deep-sea sediments. Earth Planet Sci Lett.

[CR116] Nishida N, Kazaoka O, Izumi K, Suganuma Y, Okada M, Yoshida T, Ogitsu I, Nakazato H, Kameyama S, Kagawa A, Morisaki M, Nirei H (2016). Sedimentary processes and depositional environments of a continuous marine succession across the Lower–Middle Pleistocene boundary: Kokumoto Formation, Kazusa Group, central Japan. Quat Int.

[CR117] Nomade S, Bassinot F, Marino M, Simon Q, Dewilde F, Maiorano P, Isguder G, Blamart D, Girone A, Scao V, Pereira A, Toti F, Bertini A, Combourieu-Nebout N, Peral M, Bourles DL, Petrosino P, Gallicchio S, Ciaranfi N (2019). High-resolution foraminifer stable isotope record of MIS 19 at Montalbano Jonico, southern Italy: A window into Mediterranean climatic variability during a low-eccentricity interglacial. Quat Sci Rev.

[CR118] Nowaczyk NR, Haltia EM, Ulbricht D, Wennrich V, Sauerbrey MA, Rosén P, Vogel H, Francke A, Meyer-Jacob C, Andreev AA, Lozhkin AV (2013). Chronology of Lake El’gygytgyn sediments – a combined magnetostratigraphic, palaeoclimatic and orbital tuning study based on multi-parameter analyses. Clim Past.

[CR119] Obase T, Abe-Ouchi A (2019) Abrupt Bølling-Allerød warming simulated under gradual forcing of the last deglaciation. Geophys Res Lett 46:11,397–11,405. 10.1029/2019GL084675

[CR120] Okada M, Suganuma Y (2018). Report on the PO-5 field trip to the GSSP candidate for the Middle Pleistocene Subseries on the Yoro River (Chiba Section, Japan), held during the XIX INQUA Congress 2015, 3–4 August 2015. Quat Perspect.

[CR121] Okada M, Suganuma Y, Haneda Y, Kazaoka O (2017). Paleomagnetic direction and paleointensity variations during the Matuyama-Brunhes polarity transition from a marine succession in the Chiba composite section of the Boso Peninsula, central Japan. Earth Planets Space.

[CR122] Opdyke ND, Glass B, Hays JD, Foster J (1966). Paleomagnetic study of Antarctic deep-sea cores. Science.

[CR123] Osborn HF (1900). The geological and faunal relations of Europe and America during the Tertiary Period and the theory of the successive invasions of an African fauna. Sci New Ser.

[CR124] Past Interglacials Working Group of PAGES (2016). Interglacials of the last 800,000 years. Rev Geophys.

[CR125] Pedro JB, Jochum M, Buizert C, He F, Barker S, Rasmussen SO (2018). Beyond the bipolar seesaw: Toward a process understanding of interhemispheric coupling. Quat Sci Rev.

[CR126] Penck A, Brückner E (1909). Die Alpen im Eiszeitalter.

[CR127] Peng X, Ao H, Xiao G, Qiang X, Sun Q (2020). The Early-Middle Pleistocene transition of Asian summer monsoon. Palaeogeogr Palaeoclimatol Palaeoecol.

[CR128] Pillans B (2003). Subdividing the Pleistocene using the Matuyama–Brunhes boundary (MBB): an Australasian perspective. Quat Sci Rev.

[CR129] Pinti DL, Rouchon V, Quidelleur X, Gillot P-Y, Chiesa S, Ravazzi C (2007) Comment: “Tephrochronological dating of varved interglacial lake deposits from Piànico-Sèllere (Southern Alps, Italy) to around 400 ka” by Achim Brauer, Sabine Wulf, Clara Mangili and Andrea Moscariello, Journal of Quaternary Science 22: 85–96. J Quat Sci 22(4):411–414

[CR130] Pinti DL, Quidelleur X, Chiesa S, Ravazzi C, Gillot P-Y (2001). K-Ar dating of an early Middle Pleistocene distal tephra in the interglacial varved succession of Piànico-Sèllere (Southern Alps, Italy). Earth Planet Sci Lett.

[CR131] Pol K, Masson-Delmotte V, Johnsen S, Bigler M, Cattani O, Durand G, Falourd S, Jouzel J, Minster B, Parrenin F, Ritz C, Steen-Larsen HC, Stenni B (2010). New MIS 19 EPICA Dome C high-resolution deuterium data: hints for a problematic preservation of climate variability in the “oldest ice”. Earth Planet Sci Lett.

[CR132] Prell WL, Imbrie J, Martinson DG, Morley JJ, Pisias NG, Shackleton NJ, Streeter HF (1986). Graphic correlation of oxygen isotope stratigraphy. Application to the Late Quaternary. Paleoceanography.

[CR133] Prokopenko AA, Hinnov LA, Williams DF, Kuzmin MI (2006). Orbital forcing of continental climate during the Pleistocene: a complete astronomically tuned climatic record from Lake Baikal, SE Siberia. Quat Sci Rev.

[CR134] Qiu B, Cochran SJK, Bokuniewicz HJ, Yager PL (2019). Kuroshio and Oyashio currents. Encyclopedia of Ocean Sciences.

[CR135] Quivelli O, Marino M, Rodrigues T, Girone A, Maiorano P, Abrantes F, Salgueiro E, Bassinot F (2020). Surface and deep water variability in the Western Mediterranean (ODP Site 975) during insolation cycle 74: High-resolution calcareous plankton and molecular biomarker signals. Palaeogeogr Palaeoclimatol Palaeoecol.

[CR136] Railsback LB, Gibbard PL, Head MJ, Voarintsoa NRG, Toucanne S (2015). An optimized scheme of lettered marine isotope substages for the last 1.0 million years, and the climatostratigraphic nature of isotope stages and substages. Quat Sci Rev.

[CR137] Regattieri E, Giaccio B, Mannella G, Zanchetta G, Nomade S, Tognarelli A, Perchiazzi N, Vogel H, Boschi C, Drysdale RN, Wagner B, Gemelli M, Tzedakis PC (2019). Frequency and dynamics of millennial-scale variability during Marine Isotope Stage 19: Insights from the Sulmona Basin (central Italy). Quat Sci Rev.

[CR138] Remane J, Bassett MG, Cowie JW, Gohrbandt KH, Lane HR, Michelsen O, Wang N, with the cooperation of members of ICS (1996). Revised guidelines for the establishment of global chronostratigraphic standards by the International Commission on Stratigraphy (ICS). Episodes.

[CR139] Richmond GM, Turner C (1996). The INQUA-approved provisional Lower–Middle Pleistocene boundary. The early Middle Pleistocene in Europe.

[CR140] Roberts AP, Winklhofer M (2004). Why are geomagnetic excursions not always recorded in sediments? Constraints from post-depositional remanent magnetization lock-in modeling. Earth Planet Sci Lett.

[CR141] Rossi V, Azzarone M, Capraro L, Faranda C, Ferretti P, Macrì P, Scarponi D (2018). Dynamics of benthic marine communities across the Early-Middle Pleistocene boundary in the Mediterranean region (Valle di Manche, Southern Italy): Biotic and stratigraphic implications. Palaeogeogr Palaeoclimatol Palaeoecol.

[CR142] Roulleau E, Pinti DL, Rouchon V, Quidelleur X, Gillot PY (2009). Tephro-chronostratigraphy of the lacustrine interglacial record of Piànico, Italian Southern Alps: Identifying the volcanic sources using radiogenic isotopes and trace elements. Quat Int.

[CR143] Sagnotti L, Cascella A, Ciaranfi N, Macrì P, Maiorano P, Marino M, Taddeucci J (2010). Rock magnetism and palaeomagnetism of the Montalbano Jonico section (Italy): evidence for late diagenetic growth of greigite and implications for magnetostratigraphy. Geophys J Int.

[CR144] Sagnotti L, Giaccio B, Liddicoat JC, Caricchi C, Nomade S, Renne PR (2018). On the reliability of the Matuyama–Brunhes record in the Sulmona Basin—Comment to ‘A reappraisal of the proposed rapid Matuyama–Brunhes geomagnetic reversal in the Sulmona Basin, Italy’ by Evans and Muxworthy (2018). Geophys J Int.

[CR145] Sagnotti L, Giaccio B, Liddicoat JC, Nomade S, Renne PR, Scardia G, Sprain CJ (2016) How fast was the Matuyama-Brunhes geomagnetic reversal? A new sub-centennial record from the Sulmona Basin, central Italy. Geophys J Int 204:798–812

[CR146] Sagnotti L, Scardia G, Giaccio B, Liddicoat JC, Nomade S, Renne PR, Sprain CJ (2014). Extremely rapid directional change during Matuyama-Brunhes geomagnetic polarity reversal. Geophys J Int.

[CR147] Salvador A (ed) (1994) International Stratigraphic Guide: a guide to stratigraphic classification, terminology, and procedure, 2nd edn. International Subcommission on Stratigraphic Classification of IUGS International Commission on Stratigraphy and The Geological Society of America, Boulder, p xix + 214

[CR148] Sánchez Goñi MF, Desprat S, Fletcher WJ, Morales-Molino C, Naughton F, Oliveira D, Urrego DH, Zorzi C (2018). Pollen from the deep-sea: A breakthrough in the mystery of the Ice Ages. Front Plant Sci.

[CR149] Sánchez-Goñi MF, Ferretti P, Polanco-Martínez JM, Rodrigues T, Alonso-García M, Rodríguez-Tovar FJ, Dorador J, Desprat S (2019). Pronounced northward shift of the westerlies during MIS 17 leading to the strong 100-kyr ice age cycles. Earth Planet Sci Lett.

[CR150] Sánchez-Goñi MF, Rodrigues T, Hodell DA, Polanco-Martinez JM, Alonso-Garcia M, Hernandez-Almeida I, Desprat S, Ferretti P (2016). Tropically-driven climate shifts in southwestern Europe during MIS 19, a low eccentricity interglacial. Earth Planet Sci Lett.

[CR151] Scardia G, Muttoni G (2009). Paleomagnetic investigations on Pleistocene lacustrine sequence of Piànico-Sèllere (northern Italy). Quat Int.

[CR152] Schlitzer R (2015). Ocean data view.

[CR153] Schneider T, Bischoff T, Haug GH (2014). Migrations and dynamics of the intertropical convergence zone. Nature.

[CR154] Shackleton NJ (1969). The last interglacial in the marine and terrestrial record. Proc R Soc Lond B.

[CR155] Shackleton NJ (2006). Formal Quaternary stratigraphy – What do we expect and need?. Quat Sci Rev.

[CR156] Shackleton NJ, Hall MA, Vincent E (2000). Phase relationships between millennial scale events 64,000–24,000 years ago. Paleoceanogr.

[CR157] Shackleton NJ, Opdyke ND (1973). Oxygen isotope and palaeomagnetic stratigraphy of equatorial Pacific Core V28-238: Oxygen isotope temperatures and ice volumes on a 10^5^ year and 10^6^ year scale. Quat Res.

[CR158] Shackleton NJ, Sánchez-Goñi MF, Pailler D, Lancelot Y (2003). Marine Isotope Substage 5e and the Eemian Interglacial. Glob Planet Chang.

[CR159] Simon Q, Bourlès DL, Bassinot F, Nomade S, Marino M, Ciaranfi N, Girone A, Maiorano P, Thouveny N, Choy S, Dewilde F, Scao V, Isguder G, Blamart D, Team ASTER (2017). Authigenic ^10^Be/^9^Be ratio signature of the Matuyama–Brunhes boundary in the Montalbano Jonico marine succession. Earth Planet Sci Lett.

[CR160] Simon Q, Suganuma Y, Okada M, Haneda Y, Team ASTER (2019). High-resolution ^10^Be and paleomagnetic recording of the last polarity reversal in the Chiba composite section: Age and dynamics of the Matuyama–Brunhes transition. Earth Planet Sci Lett.

[CR161] Singer BS, Jicha BR, Mochizuki N, Coe RS (2019). Synchronizing volcanic, sedimentary, and ice core records of Earth’s last magnetic polarity reversal. Sci Adv.

[CR162] Stocker TF, Johnsen SJ (2003). A minimum thermodynamic model for the bipolar seesaw. Paleoceanography.

[CR163] Studer AS, Sigman DM, Martínez-García A, Thöle LM, Michel E, Jaccard SL, Lippold JA, Mazaud A, Wang XT, Robinson LF, Adkins JF, Haug GH (2018). Increased nutrient supply to the Southern Ocean during the Holocene and its implications for the pre-industrial atmospheric CO_2_ rise. Nat Geosci.

[CR164] Suganuma Y, Haneda Y, Kameo K, Kubota Y, Hayashi H, Itaki T, Okuda M, Head MJ, Sugaya M, Nakazato H, Igarashi A, Shikoku K, Hongo M, Watanabe M, Satoguchi Y, Takeshita Y, Nishida N, Izumi K, Kawamura K, Kawamata M, Okuno J, Yoshida T, Ogitsu I, Yabusaki H, Okada M (2018). Paleoclimatic and paleoceanographic records of Marine Isotope Stage 19 at the Chiba composite section, central Japan: A reference for the Early–Middle Pleistocene boundary. Quat Sci Rev.

[CR165] Suganuma Y, Head MJ, Sagawa T, eds (2021) Stratigraphy and paleoclimatic/paleoenviromental evolution across the Early–Middle Pleistocene transition in the Chiba composite section, Japan, and other reference sections in East Asia. Prog Earth Planet Sci (this issue).

[CR166] Suganuma Y, Okada M, Head MJ, Kameo K, Haneda Y, Hayashi H, Irizuki T, Itaki T, Izumi K, Kubota Y, Nakazato H, Nishida N, Okuda M, Satoguchi Y, Simon Q, Takeshita Y, the Chiba composite section community members (in press) Formal ratification of the Global Boundary Stratotype Section and Point (GSSP) for the Chibanian Stage and Middle Pleistocene Subseries of the Quaternary System: the Chiba Section, Japan. Episodes. 10.18814/epiiugs/2020/020080

[CR167] Suganuma Y, Okada M, Horie K, Kaiden H, Takehara M, Senda R, Kimura J, Haneda Y, Kawamura K, Kazaoka O, Head MJ (2015). Age of Matuyama–Brunhes boundary constrained by U-Pb zircon dating of a widespread tephra. Geology.

[CR168] Suganuma Y, Okuno J, Heslop D, Roberts AP, Yamazaki T, Yokoyama Y (2011). Post-depositional remanent magnetization lock-in for marine sediments deduced from ^10^Be and paleomagnetic records through the Matuyama–Brunhes boundary. Earth Planet Sci Lett.

[CR169] Suganuma Y, Yokoyama Y, Yamazaki T, Kawamura K, Horng C-S, Matsuzaki H (2010). ^10^Be evidence for delayed acquisition of remanent magnetization in marine sediments: Implication for a new age for the Matuyama–Brunhes boundary. Earth Planet Sci Lett.

[CR170] Sugimoto S, Hanawa K (2009). Decadal and interdecadal variations of the Aleutian low activity and their relation to upper oceanic variations over the North Pacific. J Meteorological Soc Japan.

[CR171] Takao A, Nakamura K, Takaoka S, Fuse M, Oda Y, Shimano Y, Nishida N, Ito M (2020). Spatial and temporal variations in depositional systems in the Kazusa Group: insights into the origins of deep-water massive sandstones in a Pleistocene forearc basin on the Boso Peninsula, Japan. Prog Earth Planet Sci.

[CR172] Takeshita Y, Matsushima N, Teradaira H, Uchiyama T, Kumai H (2016). A marker tephra bed close to the Middle Pleistocene boundary: distribution of the Ontake-Byakubi tephra in central Japan. Quat Int.

[CR173] Thompson DWJ, Wallace JM (1998). The Arctic Oscillation signature in the wintertime geopotential height and temperature fields. Geophys Res Lett.

[CR174] Toti F, Bertini A, Girone A, Marino M, Maiorano P, Bassinot F, Combourieu-Nebout N, Nomade S, Buccianti A (2020). Marine and terrestrial climate variability in the western Mediterranean Sea during marine isotope stages 20 and 19. Quat Sci Rev.

[CR175] Trotta S, Marino M, Maiorano P, Girone A (2019). Climate variability through MIS 20-MIS 19 in core KC01B, Ionian Basin (central Mediterranean Sea). Alp Med Quat.

[CR176] Tzedakis PC (2010). The MIS 11–MIS 1 analogy, southern European vegetation atmospheric methane and the early anthropogenic hypothesis. Clim Past.

[CR177] Tzedakis PC, Channell JET, Hodell DA, Kleiven HF, Skinner LC (2012). Determining the natural length of the current interglacial. Nat Geosci.

[CR178] Tzedakis PC, Wolff EW, Skinner LC, Brovkin V, Hodell DA, McManus JF, Raynaud D (2012). Can we predict the duration of an interglacial?. Clim Past.

[CR179] Valet J-P, Bassinot F, Simon Q, Savranskaia T, Thouveny N, Bourlés DL, Villedieu A (2019). Constraining the age of the last geomagnetic reversal from geochemical and magnetic analyses of Atlantic, Indian, and Pacific Ocean sediments. Earth Planet Sci Lett.

[CR180] van Kolfschoten T (2020) Letter from Professor Thijs van Kolfschoten, President, INQUA to Professor Dr. Qiuming Cheng, President, IUGS; dated January 21, 2020. SQS website, http://quaternary.stratigraphy.org

[CR181] Vavrus SJ, He F, Kutzbach JE, Ruddiman WF, Tzedakis PC (2018). Glacial inception in Marine Isotope Stage 19: An orbital analog for a natural Holocene climate. Sci Rep.

[CR182] Wang Y, Yang X, Hu J (2016). Position variability of the Kuroshio Extension sea surface temperature front. Acta Oceanol Sin.

[CR183] Watkins ND (1972). Review of the development of the geomagnetic polarity time scale and discussion of prospects for its finer definition. Geol Soc Am Bull.

[CR184] Wei Y, Zhang R-H, Wang H (2017). Mesoscale wind stress–SST coupling in the Kuroshio extension and its effect on the ocean. J Oceanogr.

[CR185] Wennrich V, Minyuk PS, Borkhodoev V, Francke A, Ritter B, Nowaczyk NR, Sauerbrey MA, Brigham-Grette J, Melles M (2014). Pliocene to Pleistocene climate and environmental history of Lake El’gygytgyn, Far East Russian Arctic, based on high-resolution inorganic geochemistry data. Clim Past.

[CR186] Westerhold T, Marwan N, Drury AJ, Liebrand D, Agnini C, Anagnostou E, Barnet JSK, Bohaty SM, De Vleeschouwer D, Florindo F, Frederichs T, Hodell DA, Holbourn AE, Kroon D, Lauretano V, Littler K, Lourens LJ, Lyle M, Pälike H, Röhl U, Tian J, Wilkens RH, Wilson PA, Zachos JC (2020). An astronomically dated record of Earth’s climate and its predictability over the last 66 million years. Science.

[CR187] Wie J, Moon B-K, Lee H (2019). Variation of the relationship between Arctic Oscillation and East Asian winter monsoon in CCSM3 simulation. J Korean Earth Sci Soc.

[CR188] Yin QZ (2013). Insolation-induced mid-Brunhes transition in Southern Ocean ventilation and deep-ocean temperature. Nature.

[CR189] Yin QZ, Berger A (2015). Interglacial analogues of the Holocene and its natural near future. Quat Sci Rev.

[CR190] Yu P, Zhang L, Liu M, Zhong Q, Zhang Y, Li X (2020). A comparison of the strength and position variability of the Kuroshio Extension SST front. Acta Oceanol Sin.

[CR191] Zeileis A, Kleiber C, Krämer W, Hornik K (2003). Testing and dating of structural changes in practice. Comput Stat Data Anal.

[CR192] Zeileis A, Leisch F, Hornik K, Kleiber C (2002). Strucchange: an R package for testing for structural change in linear regression models. J Stat Softw.

[CR193] Zeuner FE (1935). The Pleistocene chronology of Central Europe. Geol Mag.

[CR194] Zeuner FE (1945). The Pleistocene Period: Its climate, chronology and faunal successions. Ray Soc London.

[CR195] Zhang Y, Wu N, Li F, Hao Q, Dong Y, Zhang D, Lu H (2020). Eco-environmental changes in the Chinese Loess Plateau during low-eccentricity interglacial Marine Isotope Stage 19. Sci China Earth Sci.

